# Correction: Genetic variations associated with adaptation in Acrocomia palms: A comparative study across the Neotropics for crop improvement

**DOI:** 10.1371/journal.pone.0333050

**Published:** 2025-09-23

**Authors:** The PLOS One Staff

## Notice of Republication

This article was republished on September 9th, 2025, to correct an error in which Table 1 was displayed as Table 2 and vice versa.

The publisher apologizes for the error. Please download this article again to view the correct version. The originally published, uncorrected article and the republished, corrected articles are provided here for reference.

## Supporting information

S1 File Originally published, uncorrected article. (PDF)

S2 File Republished, corrected article. (PDF)

## References

[1] Morales-Marroquín JA, Alves-Pereira A, Díaz-Hernández BG, Alves Vianna S, de Araújo Batista CE, Colombo CA, et al. Genetic variations associated with adaptation in Acrocomia palms: A comparative study across the Neotropics for crop improvement. PLoS One. 2025;20(6):e0324340. doi: 10.1371/journal.pone.0324340 40512773 PMC12165397

