# Supplementary figures and images for: Genetic variations associated with adaptation in Acrocomia palms: A comparative study across the Neotropics for crop improvement
Source: PLoS One. 2025 Jun 13;20(6):e0324340. doi: 10.1371/journal.pone.0324340 (PMC12165397; doi:10.1371/journal.pone.0324340)

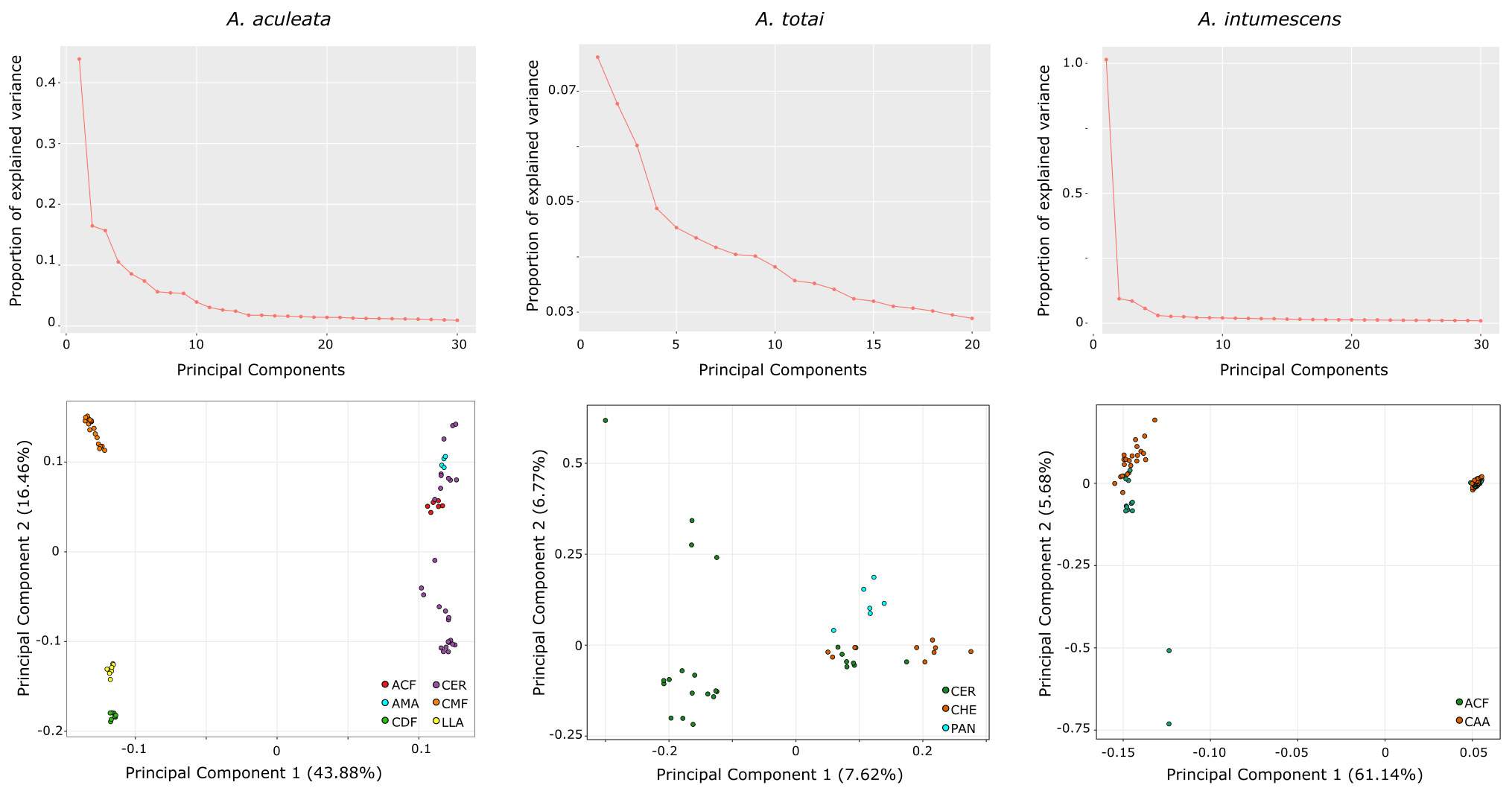

Supplement: S1 Fig — Top row: scree plots of the explained variance (y-axis) retained in each principal component (PC) (x-axis). The number of retained PCs was chosen as the first inflection point (Cattell’s rule), where the amount of genetic variation added by successive PCs reaches a plateau. Bottom row: the associated scatter plots of the first two PCs. (TIF) [file pone.0324340.s001.tif]

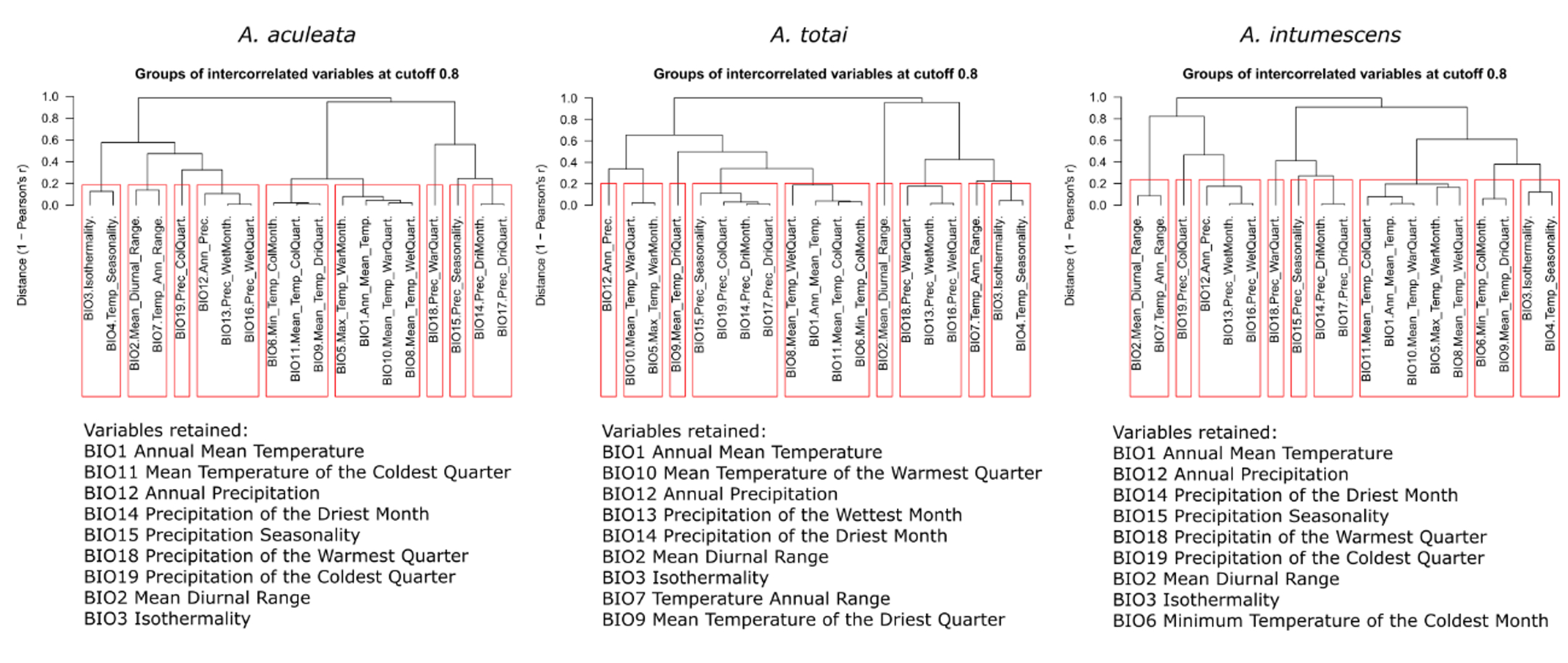

Supplement: S2 Fig — Groups of intercorrelated variables highlighted within red boxes (Pearson’s correlation coefficients > 0.8). (TIF) [file pone.0324340.s002.tif]

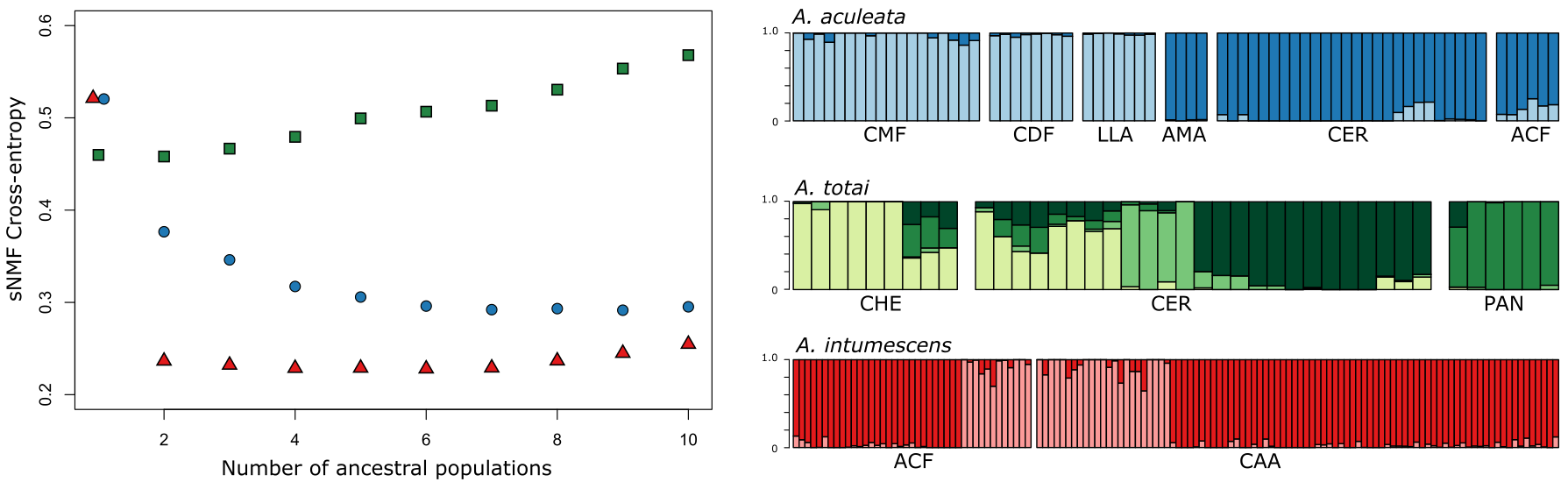

Supplement: S3 Fig — Left: plot of the cross-entropy estimates for each number of simulated ancestral populations. Right: Bar plots representing the sNMF ancestry coefficients across samples from different biogeographic group in each species. Each sample is represented by a bar and different shade colors represent their associated ancestry proportion from distinct genetic groups. Acronyms follow Table 2: CMF Central American Moist Forest; LLA Llanos; CDF Caribbean Dry Forest; ACF Atlantic Coastal Forest; CAA Caatinga; AMA Amazon; CER Cerrado; PAN Pantanal; CHE Chaco and Espinal. (TIF) [file pone.0324340.s003.tif]
