# Supplementary material for: Correction: Genetic variations associated with adaptation in Acrocomia palms: A comparative study across the Neotropics for crop improvement
Source: PLoS One. 2025 Sep 23;20(9):e0333050. doi: 10.1371/journal.pone.0333050 (PMC12456760; doi:10.1371/journal.pone.0333050)
Supplement: S1 File — (PDF) [file pone.0333050.s001.pdf]

PLOS One

RESEARCH ARTICLE

Genetic variations associated with adaptation in Acrocomia palms: A comparative study across the Neotropics for crop improvement

Jonathan A. Morales-Marroquín[1,2]*, Alessandro Alves-Pereira[2], Brenda Gabriela Díaz-Hernández[2], Suelen Alves Vianna[3], Carlos Eduardo de Araújo Batista[2], Carlos A. Colombo[3], José Baldin Pinheiro[2], Maria Imaculada Zucchi[1,2,4]*

1 Genetics and Molecular Biology Department, Biology Institute, University of Campinas (UNICAMP), Campinas, São Paulo, Brazil, **2** Department of Genetics, "Luiz de Queiroz" College of Agriculture, University of São Paulo, Piracicaba, São Paulo, Brazil, **3** Institute of Agronomy-IAC, Research Center of Plant Genetic Resources, Campinas, São Paulo, Brazil, **4** Unidade Regional de Pesquisa e Desenvolvimento (APTA), Piracicaba, São Paulo, Brazil

* jmorales1089@gmail.com (JAM-M); mizucchi@unicamp.br (MIZ)

Check for updates

OPEN ACCESS

Citation: Morales-Marroquín AJ, Alves-Pereira A, Díaz-Hernández BG, Alves Vianna S, de Araújo Batista CE, Colombo CA, et al. (2025) Genetic variations associated with adaptation in Acrocomia palms: A comparative study across the Neotropics for crop improvement. PLoS One 20(6): e0324340. https://doi.org/10.1371/journal.pone.0324340

Editor: Mozaniel Santana de Oliveira, Museu Paraense Emilio Goeldi, BRAZIL

Received: April 2, 2024

Accepted: April 23, 2025

Published: June 13, 2025

Copyright: © 2025 Morales-Marroquín et al. This is an open access article distributed under the terms of the Creative Commons Attribution License, which permits unrestricted use, distribution, and reproduction in any medium, provided the original author and source are credited.

Data availability statement: All relevant data are within the paper and its Supporting Information files. Additionally, this data set is publicly available in Figshare

Abstract

Population genetic research has evolved, focusing on selection processes using single nucleotide polymorphisms (SNP) genotyping techniques to study crop traits and domestication. This study explores the adaptation process of three neotropical palms of *Acrocomia*, a genus that has high potential for oil extraction. Our research focused on their genetic structure, evolutionary significance, and implications of the selection signatures for breeding efforts. We employed genotyping-by-sequencing (GBS) focusing on outlier SNP markers to identify adaptive genes in *A. aculeata*, *A. totai*, and *A. intumescens* across their entire distributions. Our results reveal two major gene pools in *A. aculeata*: a Central American group and a South American group, mainly influenced by dispersal and biogeographic barriers. Putative selective signatures were identified in candidate genes associated with traits related to oil biosynthesis, pathogen resistance, and adaptation to environmental stress like drought tolerance. *A. totai* exhibited a pronounced genetic structure influenced by distinct biomes, suggesting recent diversification driven by climatic and geological factors, particularly within the Pantanal biome. *A. intumescens* displays genetic structuring shaped by the endemic process of biogeographic barriers within the Caatinga biome. Correlations between allele frequencies and climatic variables highlight adaptation to diverse environments, with the annual mean temperature and precipitations being one of the most influential. Candidate genes associated with fatty acid and carotenoid biosynthesis, as well as pathogen resistance and drought tolerance, indicate targets for future breeding studies. Despite the challenges associated with reduced representation sequencing, this study highlights the potential for gene discovery in *Acrocomia*, offering promising targets to enhance oil yield productivity. Future efforts should

at the following link: https://figshare.com/s/886b6be568eae7579e4a.

Funding: We acknowledge the financial support from the São Paulo Research Foundation (FAPESP—grants:2021/10319-0, 2019/20307-0, 2014/23591-7) and the National Council for Scientific and Technological Development (CNPq—grant #431226/2018-0). We also thank the Coordination for the Improvement of Higher Education Personnel of Brazil (CAPES) for the granted scholarship Ph.D. to JM-M. Also thanks the São Paulo Research Foundation for a post-doctoral scholarship to AA-P (FAPESP 2018/00036-9). The funders had no role in study design, data collection and analysis, decision to publish, or preparation of the manuscript.

Competing interests: No authors have competing interests.

prioritize whole-genome sequencing and genotype-environment interaction studies to realize the full potential of *Acrocomia spp.* in sustainable oil production.

Introduction

The neotropical palm genus *Acrocomia* (Arecaceae: Arecoideae: Cocoseae: Bactridinae subtribe) holds significant agricultural potential. This genus comprises nine species with diverse distributions across tropical and subtropical America, and is characterized by its solitary, spiny trunk (stipe), persistent remnants of fallen leaf sheaths, and a yellow, coccoid drupe rich in oil [1]. Within the group, *Acrocomia aculeata* (Jacq.) Lodd. ex Mart. presents the widest distribution and greatest economic importance, also *A. totai* Mart. and *A. intumescens* Drude show promising potential for oil production and bioeconomy [2]. These species are popularly known as macauba, coyol, corozo, macaw palm (*A. aculeata*), bocaiúva (*A. totai*), and, macaíba (*A. intumescens*) [3]. It is worth mentioning that *A. aculeata* and the African oil palm (*Elaeis guineensis* Jacq.) exhibit analogous oil yields and fatty acid compositions [4].

A. aculeata, *A. totai*, and *A. intumescens* can be distinguishable by their distribution and distinct trunk (stipe) traits (Figs 1 and 2). Wild populations of these palms have exhibited genetic and phenotypic variations linked to environmental adaptability [5–7]. *A. aculeata* is characterized by its spiky trunk and persistent leaf bases from fallen leaves. The total yield obtained (dry basis) in the extraction of *A. aculeata* fruit pulp oil was 65% w/w from populations of the Brazilian state of Minas Gerais [4], and 53.6% from populations of Costa Rica [8]. Its distribution ranges from the subtropical to tropical regions of northern Mexico through Central America (Guatemala, El Salvador, Honduras, Nicaragua, Costa Rica, and Panama), Caribbean Islands, into Colombia, Venezuela, Guyana, Brazil, and northern Argentina [1]. It is present in six of the seven proposed neotropical palm biogeographic areas, predominantly in transitions between macro ecoregions, i.e.,: tropical rainforests (moist broadleaf forest) to drier ecosystems like savannas, grasslands, dry broadleaf forests, and xeric shrublands (Fig 2) [9,10]. It occurs in the following local biomes: Brazilian Cerrado, Atlantic coastal forest, Amazon, Central America moist forest, Caribbean dry forests, and Llanos [11,12].

A. totai generally has a smooth trunk, occasionally with spikes, and rarely covered in persistent leaf bases. The total yield obtained (dry basis) in the extraction of *A. totai* pulp oil was 34.7% w/w from populations of the Brazilian state of Mato Grosso do Sul [13]. It is distributed across a preeminent subtropical region in southern Brazil, northern Argentina, Bolivia, and Paraguay, occurring in the local biomes of Brazilian Pantanal, Chaco, and Espinal, as well as the Brazilian Cerrado transition biome. In macro-ecoregion classifications, it occurs in moist broadleaf forests, flooded grasslands, and savannas, as well as in drier ecosystems such as savannas, grasslands, and shrublands (Fig 2) [10,12]. *A. intumescens* has a swollen trunk with spikes when young, which later becomes smooth in adulthood (Fig 1) [1]. The total yield obtained (dry basis) in the extraction of *A. intumescens* pulp oil was 26.9% w/w from populations of the Brazilian state of Pernambuco [14]. *A. intumescens* has a more restricted

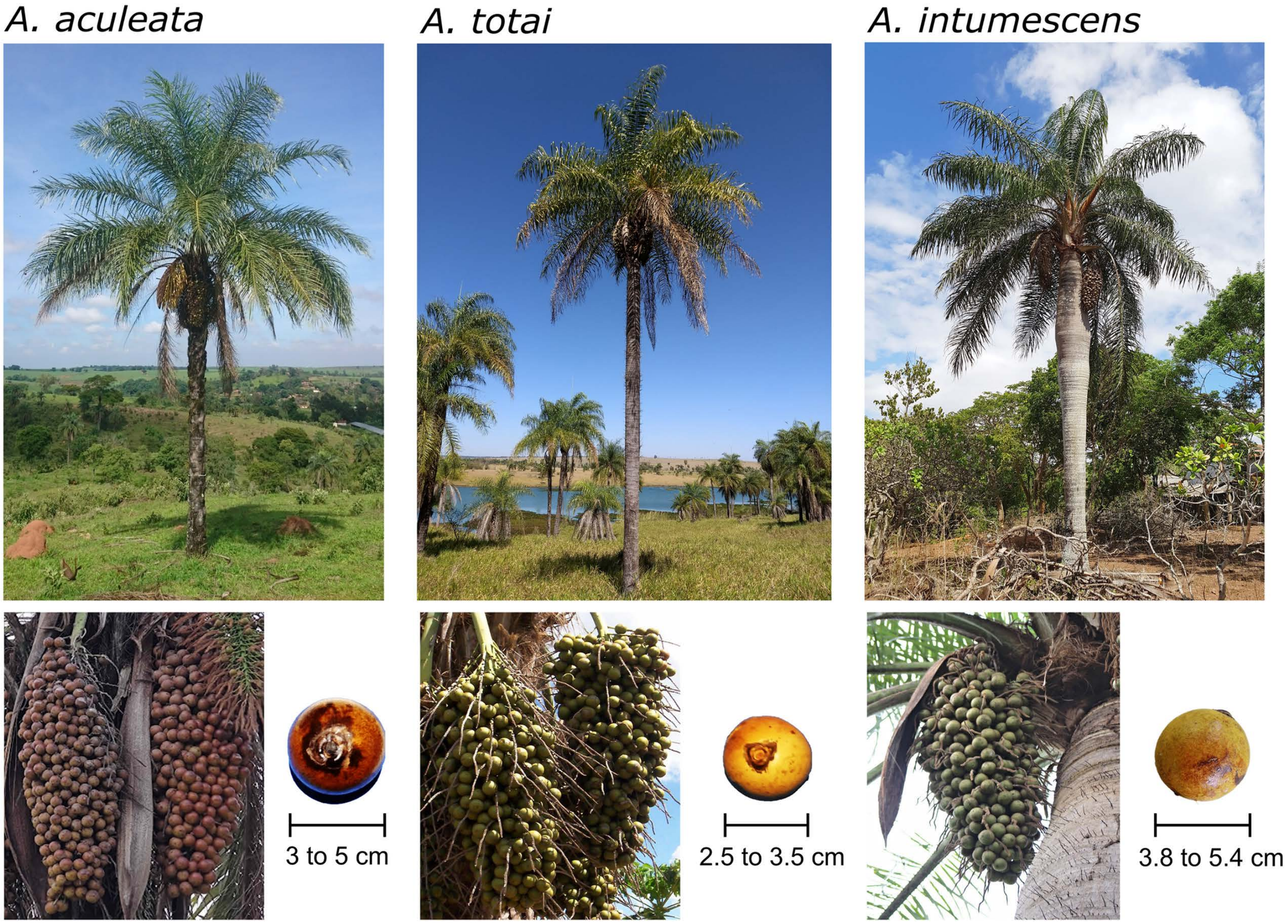

Fig 1. Adult plants of *Acrocomia aculeata*, *Acrocomia totai*, *Acrocomia intumescens*, and their fruits. Location: *A. aculeata* – Palm from Itapira, São Paulo, Brazil. Fruits from Minas Gerais, Brazil. *A. totai* – Palm from Presidente Epitácio, São Paulo, Brazil. Fruits from Corumbá, Mato Grosso do Sul, Brazil. *A. intumescens* – Palm and fruits from Vale da Neblina, Paraíba, Brazil. Photo credits: Brenda Díaz-Hernández (Palms: *A. aculeata* and *A. totai*), Suelen Alves Vianna (Fruits: *A. aculeata* and *A. totai*), and Eulampio Duarte (*A. intumescens* Palm and Fruits).

https://doi.org/10.1371/journal.pone.0324340.g001

distribution, endemic to the arid and semi-arid tropical regions of the transition zones between the local biomes of Brazilian Caatinga and Cerrado, also in Atlantic coastal forest in the northeastern states of Pernambuco, Ceará, Paraíba, and Maranhão [11]. In macro-ecoregion classifications, it occurs in dry broadleaf forest (Fig 2) [10].

A. aculeata, or macaúba, is increasingly recognized as a promising crop, offering a diverse array of emerging high-value commodities. These include products derived from its fruits, leaves and their press cakes as well as high-content oils suitable for various applications including biofuels, human and animal consumption (vegetable oil and flours), cosmetics, and pharmaceutics. Furthermore, *A. aculeata* holds significant promise for sustainable biomass production, raising important considerations regarding land use, biodiversity conservation, carbon sequestration and storage, and its potential contributions to climate and environmental protection [2,4]. Biofuels derived from *Acrocomia* could facilitate the transition from fossil fuels to more sustainable energy options in both, developed and developing countries. The promotion of macauba as a crop suitable for cultivation in semi-arid environments, including rangeland, bare ground, degraded cropland, or grassland, aligns with multiple United Nations Sustainable Development Goals (SDGs): (1) SDG 7 (Affordable

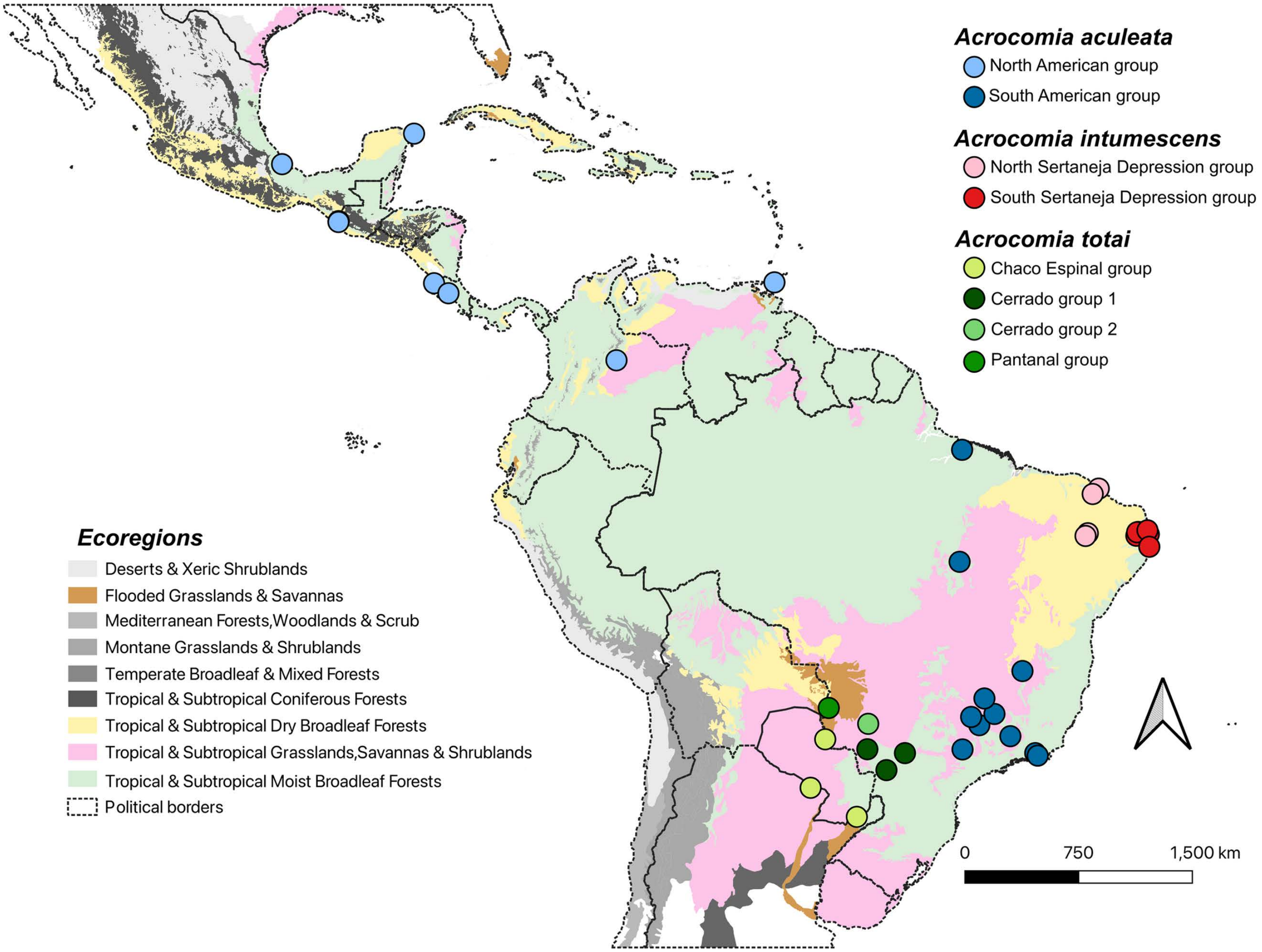

Fig 2. Map showing sampling locations of *Acrocomia* natural populations in the various macro ecoregions of the neotropics. The map displays populations and major genetic groups within them of *Acrocomia aculeata* (blue dots), *A. totai* (green dots), and *A. intumescens* (red dots), as described in Table 2. The different macro ecoregions of the neotropics are defined according to Dinerstein et al. [10]. Shapefile of limits of countries in South America from IBGE-Mapas (IBGE—Brazilian Institute of Geography and Statistics; Available at: https://geoftp.ibge.gov.br/cartas_e_mapas/bases_cartograficas_continuas/bc250/versao2021/shapefile/bc250_shapefile_2021_11_18.zip) The map was developed by the authors using QGIS Version 3.32.3 (Available at: <https://qgis.org/en/site/>). DATUM: SIRGAS 2000.

https://doi.org/10.1371/journal.pone.0324340.g002

and Clean Energy) by providing a renewable fuel source, (2) SDG 13 (Climate Action) by potentially mitigating carbon emissions and promoting climate-resilient agriculture, and (3) SDG 15 (Life on Land) by restoring degraded land and potentially mitigating biodiversity loss.

These three species easily occur in anthropized areas, and their dispersion is favored by human interaction [4]. There is evidence that *Acrocomia* was used by Pre-Columbian civilizations as a ritual and edible plant [15–17]. The oldest archaeological record of its utilization in Central America is dated from the Pre-Olmec and Olmec civilizations in the southern Mexican state of Veracruz (4000 BP) [18]. The oldest human interaction in South America is with the Amerindians and other proto-Tupi-Guarani civilizations in the Brazilian lowlands dated 10,030 BP. The oldest known dated fossil

was described in the northern Brazilian state of Pará (Santarém – 11,200 BP), followed by Argentina (8500 BP), Panama (8040 BP), and Mexico (6750 BP) [1,19]. *A. aculeata* is considered an incipiently domesticated crop and probably share the same evolutionary pressures as other domesticated neotropical crops for new ecological niche of cultivation and adaptation [17]. Despite the growing economic interest in some *Acrocomia* species, understanding of their genomic information remains limited, especially adaptation strategies and gene flow relationships.

The development of new crop varieties with improved traits such as disease resistance, drought tolerance, and high oil yield can be achieved through the identification of local varieties (ecotypes), genetic markers, and quantitative trait loci (QTL) mapping [20,21]. Understanding the genetic diversity and potential gene targets of *Acrocomia* palms is essential for identifying desirable characteristics and selecting suitable breeding materials to enhance oil yield productivity. The ability of crop species to adapt and maintain resilience in response to changing environmental conditions is significantly influenced by genetic diversity [22]. Using loci under selection, breeders can identify and characterize candidate genes responsible for key adaptive traits of macauba cultivars [23]. Furthermore, this research can provide insights on the mechanisms underlying *Acrocomia* diversification in the neotropics by identifying important evolutionary processes such speciation, adaptation, and dispersal.

Despite growing interest in macauba as a promising crop for semi-arid and degraded lands, significant gaps remain in understanding the genetic mechanisms underlying its adaptation to these challenging environments. Previous studies have been limited by a lack of high-resolution genomic data in non-model crops, limiting the identification of adaptive loci and their functional roles in environmental adaptation and oil production. Additionally, traditional breeding methods in perennial species like macauba have struggled to link genotype to phenotype for complex heterogenous adaptive traits, particularly in response to abiotic stresses such as drought and soil degradation. The application of genomic tools, such as genotyping-by-sequencing (GBS) to study adaptation in tropical species like macauba has been scarce. This study assessed selective signatures and genome-wide diversity in natural populations of *A. aculeata*, *A. totai*, and *A. intumescens* from various Neotropical biomes using reduced representation sequencing. By linking genomic variation to environmental adaptation, this research not only advances our understanding of the genetic basis of climate resilience in neotropical palms but also provides actionable insights for breeding programs aimed at enhancing the sustainability of macauba cultivation in degraded and semi-arid landscapes. This research aims to contribute to the domestication and breeding of this promising tropical palm for sustainable vegetable oil production, bioeconomy, and biofuels.

Results

SNP discovery in genomic libraries of the three studied *Acrocomia* species

Sequencing of the two ddGBS libraries for *A. aculeata* and *A. totai* generated a total of 219,264,253 reads, while the two *A. intumescens* ddGBS libraries generated a total of 554,630,990 reads. After quality-control filtering, the number of retained reads were: 60,786,924 (mean = 779,319.5 reads per sample, SD ± 449,982) for *A. aculeata*; 29,283,574 (mean = 697,227.9 reads per sample, SD ± 355,813) for *A. totai*; and 276,741,447 (mean = 1,990,945.6 reads per sample, SD ± 1,333,058) for *A. intumescens*. After the analysis in Stacks, the final data set had 1997 SNPs for the 78 *A. aculeata* samples (mean depth per locus = 18.7X, SD ± 6.2, 8.8% of missing data). For the 40 *A. totai* samples, 1629 SNPs were identified (mean depth per locus = 17.2X, SD ± 6.4, 2.9% of missing data). For the 131 *A. intumescens* samples a total of 3466 SNPs were identified (mean depth per locus = 11.5X, SD ± 4.4, 13.4% of missing data).

Putative signatures of selection and population structuration in *A. aculeata*, *A. totai*, and *A. intumescens*

Because pcadapt and LFMM are methods that account for the global genetic structure of the data, some interesting patterns may be considered for the analyses of putative signatures of selection. Both analyses suggested that the genetic structure within *A. aculeata* and *A. intumescens* is not explained by the local biome groups assumed according to the classification proposed by Freitas *et al*. [12], but rather the outcome of genomic isolation of biogeographic barriers and

historical events. In the case of *A. totai*, a genetic structuring was observed in accordance with local biomes and macro ecoregions (Fig 3, S1 and S3 Figs) suggesting local ecosystem adaptation. Similar DAPC results were obtained for *A. totai*, indicating a significant degree of divergence between natural populations across specific biomes. The major genetic structure in *A. aculeata* is observed among sampling locations (or biogeographic groups) within and outside Brazilian lowlands (<1500 masl), resulting in the separation of the samples into two clusters along the first principal component

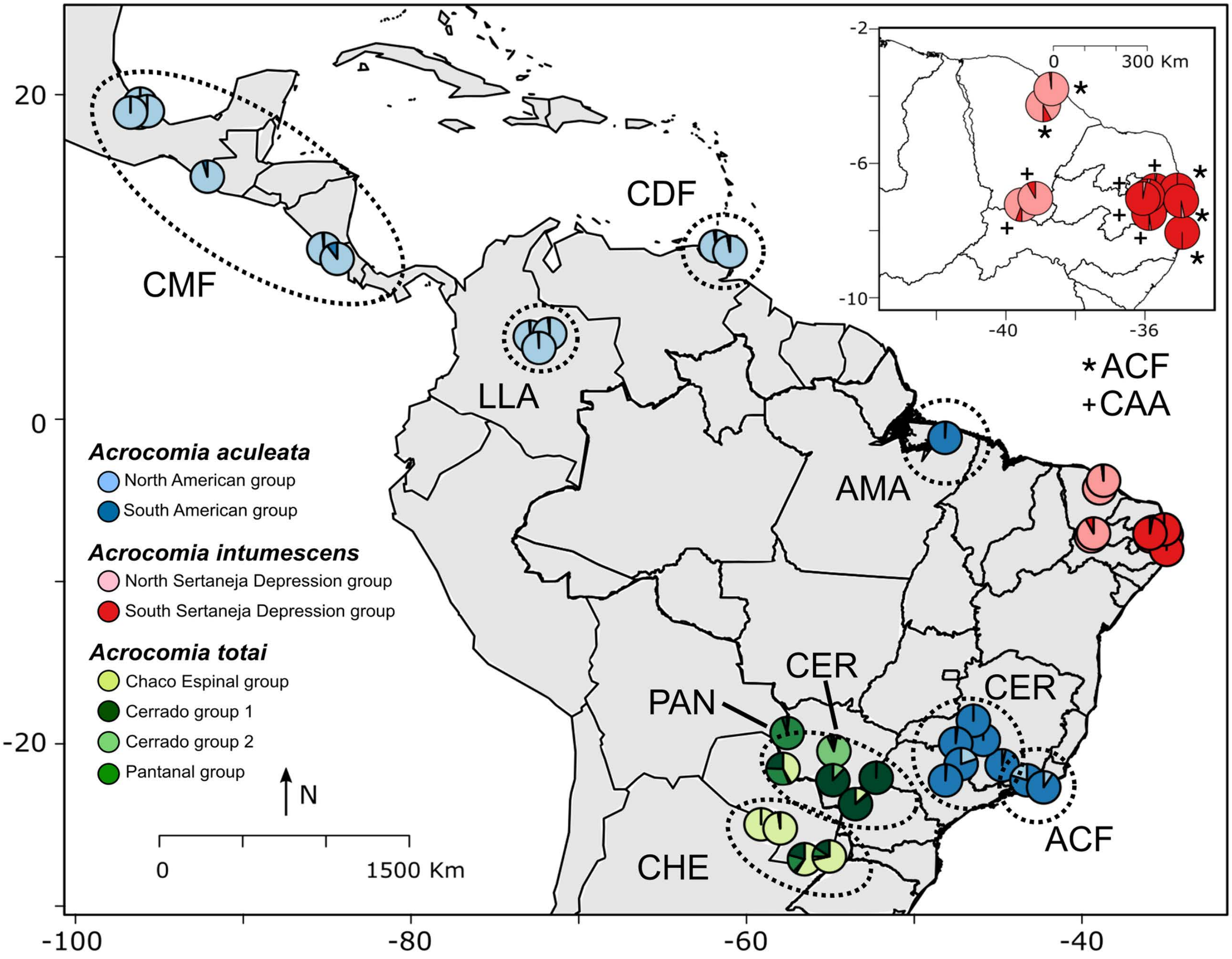

Fig 3. Map of sampling locations for *Acrocomia aculeata* (blue), *A. totai* (green), and *A. intumescens* (red), along with major genetic divergences among local biome groups as suggested by sparse non-negative matrix factorization (sNMF) analyses. Pie charts show the average sNMF ancestry coefficients across the genetic clusters represented by different color shades. Some pie charts were slightly moved to ease visualization. Acronyms follow Table 2 local biomes: CMF Central American Moist Forest; LLA Llanos; CDF Caribbean Dry Forest; ACF Atlantic Coastal Forest; CAA Caatinga; AMA Amazon; CER Cerrado; PAN Pantanal; CHE Chaco and Espinal. Shapefile of limits of countries in South America from IBGE-Mapas (IBGE—Brazilian Institute of Geography and Statistics; Available at: https://geoftp.ibge.gov.br/cartas_e_mapas/bases_cartograficas_continuas/bc250/versao2021/shapefile/bc250_shapefile_2021_11_18.zip) The map was developed by the authors using QGIS Version 3.32.3 (Available at: <https://qgis.org/en/site/>). DATUM: SIRGAS 2000.

https://doi.org/10.1371/journal.pone.0324340.g003

(43.88% of the variance), with a distinct divergence between samples from Mexico and Central America and those from Brazil (Fig 3 and S1 Fig). In *A. totai*, samples from Pantanal (PAN) were the most divergent, the Argentinean-Paraguayan Chaco and Espinal (CHE) samples tend to cluster in the same genetic group, while samples from the Brazilian Cerrado (CER) were more admixed than those from the other groups (Fig 3 and S1 Fig). The major genetic divergence in *A. intumescens* was observed between the Brazilian state of Ceará, indicating interrupted gene flow likely due to genetic isolation or a biogeographic barrier, rather than between biomes (Fig 3 and S1 Fig).

The numbers of outlier SNPs detected for *A. aculeata* were 500 based on pcadapt and 129 based on FstHet, while LFMM indicated 525 loci associated with at least one bioclimatic variable. Of these, 326 markers were considered as putative under selection because they were indicated by at least two of these methods (Fig 4A). A total of 67 *A. aculeata* sequences with an outlier marker had at least one blastx hit, and 54 had at least one GO annotation. These GO terms could be summarized in 33 classes (Fig 5A). For *A. totai*, pcadapt indicated 218 outlier SNPs, FstHet 306, and 446 SNPs were associated to a bioclimatic variable in LFMM. Of these, 192 SNPs were considered as putative under selection (Fig 4B). A total of 38 *A. totai* sequences with an outlier marker had at least one blastx hit, and 33 had at least one GO annotation. These GO terms could be summarized in 28 classes (Fig 5B). For *A. intumescens*, pcadapt indicated 688 outlier SNPs, FstHet 157, and 1024 markers were associated with a bioclimatic variable in LFMM. Of these, 104 SNPs were considered as markers putatively under selection (Fig 4C). Only eight *A. intumescens* sequences with an outlier marker had at least one blastx hit, and two had GO annotations. These GO terms could be summarized in seven classes (Fig 5C). For the three species, the most common GO annotations were related with the biological processes of "metabolic process" and "cellular process", and the molecular functions of "binding" and "catalytic activity". GO annotations related to "response to stimulus" and "regulation" were also frequent for the proteins with similarities to the sequences with outlier SNPs of *A. aculeata* and *A. totai*.

These genes exhibited high similarity to genes annotated and described in palm species such as *Elaeis guineensis*, *Phoenix dactylifera* L., and *Cocos nucifera* L., all of which have fully sequenced genomes (Table 1, S2 Table). The genes observed were related to many functions, including oil production metabolism/catabolism, carotenoid biosynthesis, plant growth and development, organ sizes, root, flowering, pathogen resistance, and biotic and abiotic stresses. The most relevant loci identified as outliers in different methods and for each species are listed in Table 1 (the whole list is in S1 Table), while a summary of blastx results and their associated GO annotations are in S2 Table.

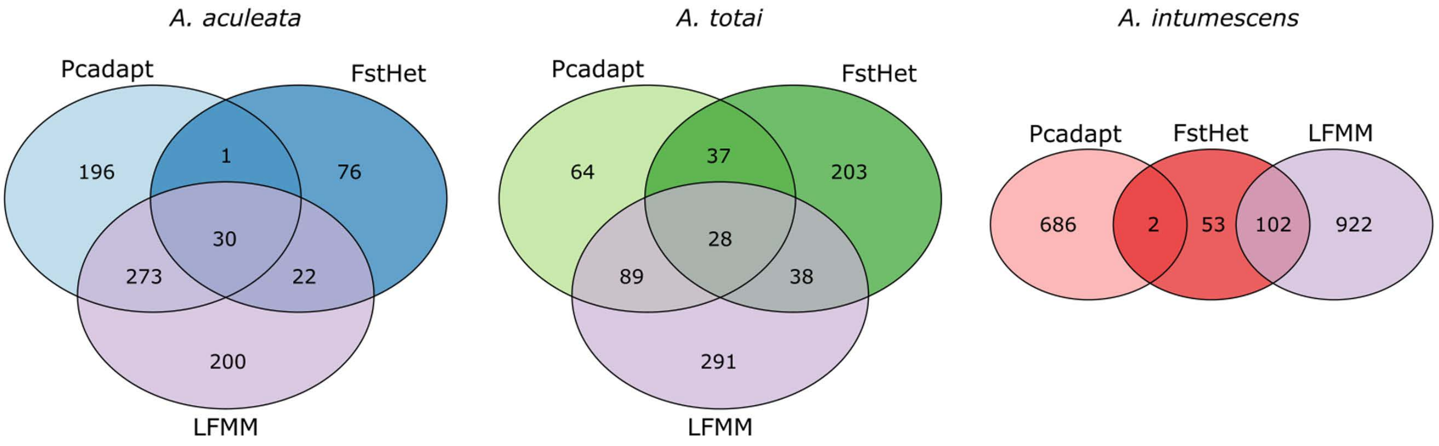

Fig 4. Venn diagrams showing the number of loci identified as outliers with different methods. The outlier SNPs detected in Pcadapt are based on the genetic structure of the PCA, while in FstHet, they are based on the betahat statistic. In LFMM, the outliers are associated with bioclimatic variables. **A)** *Acrocomia aculeata*, **B)** *A. totai*, and **C)** *A.intumescens*.

https://doi.org/10.1371/journal.pone.0324340.g004

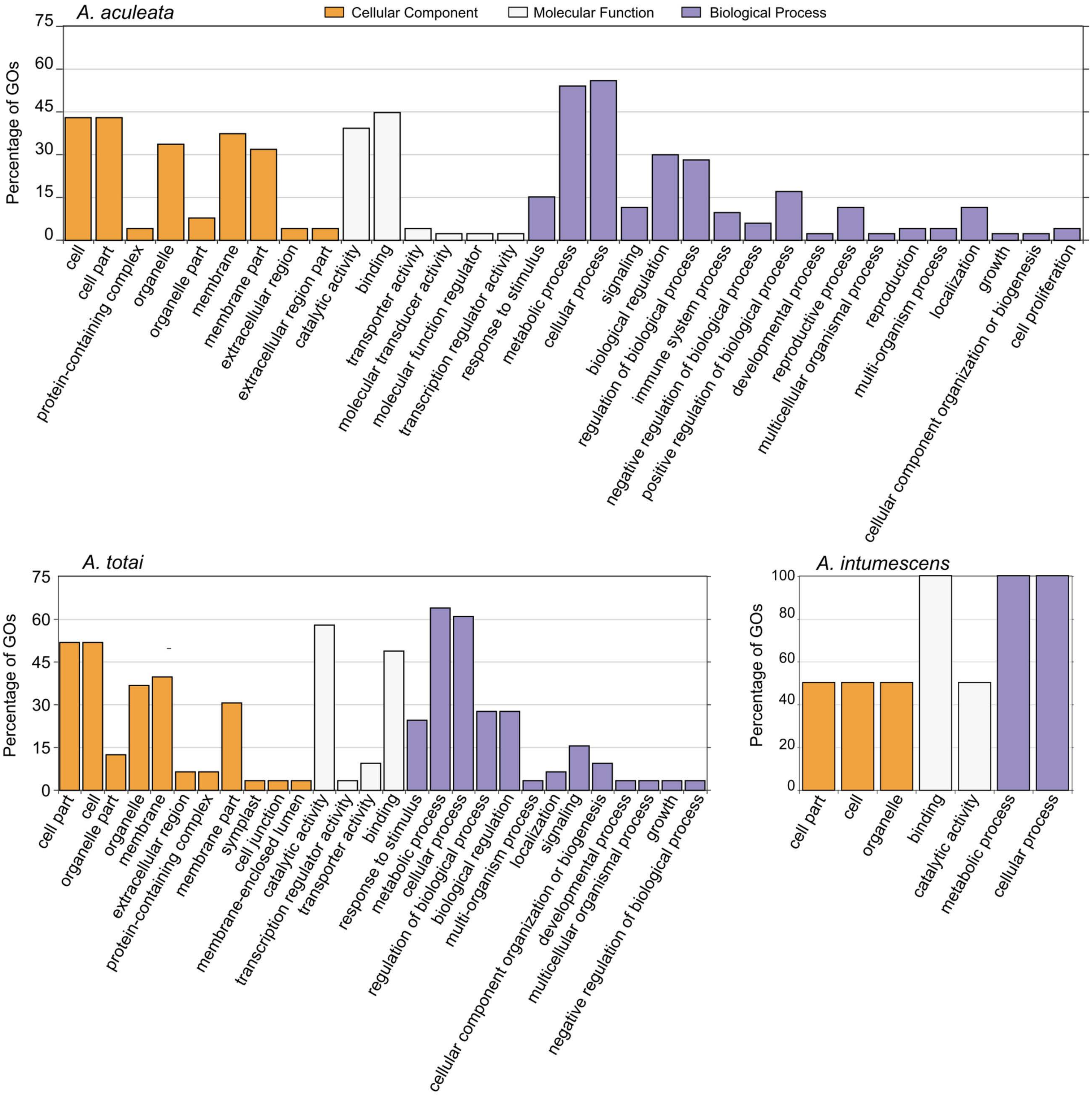

Fig 5. Summary of Gene Ontology (GO) annotations associated with proteins. The outlier SNPs in gene sequences associated with proteins are deposited in GenBank. GO terms summarize annotations according to cellular components, molecular functions, and biological processes. **A)** *Acrocomia aculeata*, **B)** *A. totai*, and **C)** *A.intumescens*.

https://doi.org/10.1371/journal.pone.0324340.g005

Table 1. Most relevant gene loci with putative selective signatures in *Acrocomia aculeata*, *A. totai*, and *A. intumescens*. Gene loci are divided into four major biological functional groups: (1) fatty acid and triacylglycerol biosynthesis, (2) carotenoid biosynthesis and plant development, (3) plant defense and disease resistance, and (4) abiotic and environmental stress. The complete list of gene loci and their description can be found in S2 Table.

Acrocomia aculeata							
**Fatty acid and triacylglycerol biosynthesis**	**Sequence name**	**Sequence desc.**	**Species**	**Function**	**E-Value**	**Similarity**	**Reference**
	CLocus_32860	ABC transporter D family member 1	*Capsicum annuum*	Lipid metabolism (long-chain fatty acid transporter activity, Long-chain fatty acid import into peroxisome; fatty-acyl-CoA transport), Organ growth, plant nutrition, plant development, response to abiotic stress, and the interaction of the plant with its environment	2.37E-04	100	[24,25]
	CLocus_25725	Lipid binding protein	*Elaeis guineensis*	Lipid biosynthesis Lipid transport; Lipid binding	2.37E-09	100	[26]
	CLocus_11291	Patatin-like protein 2	*Cocos nucifera*	Lipid Metabolism (phospholipase activity; lipid catabolic process; acylglycerol lipase activity); Resistance to pathogens	3.83E-11	96.7	[25,27]
	CLocus_24655	Pentatricopeptide repeat-containing protein At5g15340, mitochondrial	*Elaeis guineensis*	Zinc ion binding. Influence organellar expression function and, consequently, on photosynthesis, respiration, plant development, and environmental responses	9.48E-10	100	[28]
	CLocus_12388	Pentatricopeptide repeat-containing protein, mitochondrial-like	*Cocos nucifera*	Zinc ion binding. Influence organellar expression function and, environmental responses	1.20E-06	86.7	[28]
	CLocus_16644	Polyprenol reductase 2	*Phoenix dactylifera*	Essential in dolichol biosynthesis	7.80E-10	100	[29,30]
	CLocus_27100	Putative WRKY transcription factor 19	*Elaeis guineensis*	Modifies the content of fatty acids and influences the accumulation of seed oil; Abiotic stress responses, and plant pathogen interactions	1.96E-10	100	[31,32]
**Carotenoid biosynthesis & Plant development**	CLocus_16512	Carotenoid cleavage dioxygenase 7 (chloroplastic)	*Elaeis guineensis*	Carotene catabolic process, Strigolactone biosynthesis that modulates plant growth, reproduction, senescence	4.87E-09	89.7	[33–36]
	CLocus_11820	Carotenoid cleavage dioxygenase 7, chloroplastic	*Phoenix dactylifera*	Carotene catabolic process, modulates plant growth, reproduction, senescence	4.37E-09	90	[33,36,37]
	CLocus_17318	Carotenoid cleavage dioxygenase 8 homolog B (chloroplastic)	*Morus notabilis*	Apocarotenoids, carotenoid cleavage dioxygenases (CCDs), inhibit shoot branching. Also active on other carotenoid substrates like lycopene or zeaxanthin	1.92E-11	100	[38,39]
	CLocus_21286	GRF-interacting factor 1	*Elaeis guineensis*	Leaf development and growth, reproductive system development and growth	3.67E-09	96.6	[40,41]
	CLocus_18527	Protein OBERON 3	*Phoenix dactylifera*	Embryonic root meristem initiation, rooting	2.16E-10	100	[42,43]

(Continued)

Table 1. (Continued)

Acrocomia aculeata							
**Plant defense and disease resistance**	CLocus_29621	BTB/POZ domain and ankyrin repeat-containing protein NPR2	*Cocos nucifera*	Plant defense and plant disease resistance	4.59E-10	100	[44]
	CLocus_35203	BTB/POZ domain and ankyrin repeat-containing protein NPR2	*Elaeis guineensis*	Plant defense and plant disease resistance	3.68E-10	100	[44]
	CLocus_82942	Endoglucanase 8-like	*Elaeis guineensis*	Synthesis, remodeling and turnover of cell wall components in symbiotic and hostile plant–microbe interactions	1.09E-06	100	[45]
	CLocus_22454	Momilactone A synthase	*Cocos nucifera*	Momilactone A biosynthesis, diterpenoid secondary metabolites, involved in the defense mechanism of the plant produced in response to attacks	9.13E-12	100	[46,47]
	CLocus_317	Polyprenol reductase 1 isoform X1	*Elaeis guineensis*	Abiotic stress responses, and plant pathogen interactions, lipase activity	9.80E-08	96.6	[29,30]
	CLocus_30624	Subtilisin-like protease SBT1.5	*Elaeis guineensis*	Plant–pathogen recognition and immune priming	5.86E-09	96.6	[48]
**Abiotic and environmental stress**	CLocus_21703	FRIGIDA-like protein 4a	*Elaeis guineensis*	Regulates flowering induction, flowering time	2.22E-04	100	[49]
	CLocus_23382	Phosphoinositide phospholipase C 2	*Elaeis guineensis*	Signal transduction in events such as guard cell signaling, salt stress, osmotic stress, acquired resistance, Nod factor signaling, drought, systemic acquired resistance, carbon fixation in C4 plants, and response to the pathogen	4.27E-12	100	[50]
	CLocus_25856	Putative pentatricopeptide repeat-containing protein At5g37570	*Elaeis guineensis*	Organelle biogenesis and function, photosynthesis, respiration, plant development, and environmental responses	1.15E-10	96.6	[28]
Acrocomia totai							
Fatty acid and triacylglycerol biosynthesis	**Sequence name**	**Sequence desc.**	**Species**	**Function**	**E-Value**	**Similarity**	**Reference**
	CLocus_17145	GDSL esterase/lipase	*Elaeis guineensis*	Lipid metabolism: produce and storage oil in seeds, cuticular lipids to cover and decorate organ surfaces, oxylipins, and other signaling molecules	7.53E-11	100	[51,52]
	CLocus_20717	Fatty acid desaturase 4, chloroplastic	*Cocos nucifera*	Influence levels of susceptibility to multiple stresses, including insect infestations	2.21E-09	100	[53]
Carotenoid biosynthesis & Plant development	CLocus_16267	Mitogen-activated protein kinase kinase kinase NPK1	*Elaeis guineensis*	Regulates innate immunity and development in plants	1.33E-08	93.1	[54]
	CLocus_21866	Phosphatidylinositol 4-kinase alpha 1 isoform X2	*Elaeis guineensis*	Essential for pollen, embryonic, and post-embryonic development also cell signaling	5.17E-10	100	[55]
	CLocus_37352	Protein OBERON 3	*Phoenix dactylifera*	Embryonic root meristem initiation, rooting	2.21E-10	100	[42,43]

(Continued)

Table 1. (Continued)

Acrocomia aculeata							
Plant defense and disease resistance	CLocus_37219	Subtilisin-like protease SBT1.2	*Elaeis guineensis*	Plant–pathogen recognition and immune priming	3.18E-11	100	[48]
	CLocus_29376	Subtilisin-like protease SBT1.2	*Elaeis guineensis*	Plant–pathogen recognition and immune priming	2.36E-10	93.3	[48]
	CLocus_54281	Phospholipase A2-alpha	*Elaeis guineensis*	Signaling roles during plant abiotic and biotic stress responses	2.52E-09	96.6	[56]
	CLocus_12178	Protein ENHANCED DISEASE RESIS-TANCE 4	*Cocos nucifera*	Regulation of defense response to fungus	1.61E-05	93.3	[57]
Abiotic and environmental stress	CLocus_15942	Low affinity sulfate transporter 3	*Cocos nucifera*	Plant response to drought and salinity stress	7.41E-10	100	[58]
	CLocus_24114	9-cis-epoxycarotenoid dioxygenase NCED6, chloroplastic	*Cocos nucifera*	Increases drought tolerance, multi-abiotic stress tolerance, regulates plant growth, also enhances seed dormancy	3.42E-13	96.6	[59–61]
	CLocus_26989	Momilactone A synthase	*Cocos nucifera*	Drought, salinity, and oxidative stress conditions tolerance	1.58E-11	100	[62,63]
Acrocomia intumescens							
Abiotic and environmental stress	**Sequence name**	**Sequence desc.**	**Species**	**Function**	**E-Value**	**Similarity**	**Reference**
	CLo-cus_111127	Phosphatase 2C BIPP2C1	*Elaeis guineensis*	Plant signal transduction processes and stress signaling	3.23E-09	100.0	[64]

https://doi.org/10.1371/journal.pone.0324340.t002

Our comparative analysis across the three *Acrocomia* species (A. aculeata, A. totai, and A. intumescens) revealed both common and species-specific patterns in their adaptation mechanisms to environmental stressors. While all species showed significant evidence of selection in response to bioclimatic variables associated to annual temperature (BIO 1), diurnal range (BIO 2), annual precipitation and especially in the driest month (BIO 12, 14), the degree of genetic divergence and the nature of the putatively selected loci varied notably. *A. aculeata* exhibited a strong genetic structure linked to biogeographic barriers, with outlier loci primarily associated with abiotic stress responses, such as fatty acid biosynthesis and carotenoid metabolism, suggesting a robust adaptation to fluctuating environmental conditions. In contrast, *A. totai* showed more localized genetic structuring in relation to specific biomes, with genes related to lipid metabolism, plant defense, and abiotic stress tolerance playing key roles in its adaptation to different ecological zones, such as the Pantanal and Cerrado. *A. intumescens*, on the other hand, displayed a notable divergence primarily in samples from Ceará, indicating possible genetic isolation and local adaptation to unique environmental pressures. Despite these differences, all three species shared common functional pathways related to metabolic processes, cellular functions, and stress responses, such as plant defense and abiotic stress tolerance. These findings highlight the diverse adaptive strategies employed by *Acrocomia* species, emphasizing the complex interplay between genetic isolation, local environmental factors, and evolutionary pressures in shaping their genomic diversity.

Discussion

The focus of population genetic research has rapidly shifted from spatially neutral genetic processes to adaptive genetic processes, driven by advancements in SNP genotyping techniques for studying crop traits and domestication processes. Considerable deviation of outlier markers from the distribution of a specific statistical evaluation under a particular model can serve as the basis for methods aimed at detecting selective signatures for understanding adaptation events [23,65]. A significant future development in sustainable agriculture will involve the domestication of novel native plant species, such as *Acrocomia*, to facilitate the transition towards cleaner energy sources. Below, we discuss the most interesting findings regarding outlier SNPs in the adaptation process of *Acrocomia*, their biological and evolutionary significance, and their implications for breeding efforts.

The adaptation and evolutionary process in *Acrocomia*: dispersal and biogeographic barriers

Acrocomia phenotypes have been shaped by ecological interactions that have influenced the evolution of specific traits for its survival [66]. *Acrocomia* species are distributed across tropical Americas, extending from Central America (Northern Mexico) to South America, inhabiting areas transitioning from tropical and subtropical rainforests (moist broadleaf forests) to savannah and xeric shrubland regions, including dry broadleaf forests (semi-arid and arid ecosystems) (Fig 2). Their wide distribution suggests their ability to adapt to various soil and climate conditions. sNMF analyses can help identify genetic signatures associated with adaptation to specific environmental conditions or phenotypic traits. Our findings provide insights into the genetic structure and the association of specific SNP loci under selection forces in the adaptation process of *Acrocomia aculeata*, *A. totai*, and *A. intumescens* (Fig 4, Table 1).

Our results support the division of *A. aculeata* into two major gene pools: a Central American group and a South American group, consistent with previous observations based only on neutral loci [3] (Figs 2 and 3). Notably, we found no significant differences in genetic structure within minor local biomes within each gene pool, indicating that the Amazon basin likely served as a source region for diversification across the species distribution. This pattern in genetic structure is also present in other crop species such as common beans and maize [67,68]. Both dispersal and biogeographic barriers likely influenced the genomic structure of *Acrocomia aculeata*. Also, *A. aculeata* exhibited the greatest number of putative selective signatures (Table 1; S1 and S2 Tables) in genes associated with fatty acid and triacylglycerol biosynthesis, carotenoid biosynthesis, pathogen resistance and defense, as well as genes specialized in adapting to abiotic and environmental stress. From the nine species of the genus, *A. aculeata* exhibits the highest fruit pulp oil content (65% w/w dry basis) [4].

Individual plant fitness is influenced by animal–plant interactions, encompassing mutualisms such as seed dispersal and antagonistic relationships like herbivory and seed predation [66]. For example, the morphology of *Acrocomia aculeata* fruits, including its size, color, and content, appears to have undergone a correlated evolution as predicted by the seed dispersal syndrome hypothesis before the domestication process by humans began in recent times (~10,000 BP) [1,19]. Additionally, spines and other mechanical defense features are common mechanisms employed by plants to avoid herbivory. Numerous studies on palms indicate that spinescence reduces herbivory by large mammals [69]. The same herbivory and seed dispersal pattern is present in *A. totai* and *A. intumescens*.

Frugivorous mammals have a preference for consuming dull-colored fruits, such as those that are green, yellow, brown, or pale orange [66,70] (Fig 1). It is hypothesized that the coccoid type fruit (drupe) of *Acrocomia* ancestor species may have been consumed by large herbivores in the past, with migration events like the Great American Biotic Interchange (GABI; 2.5 million BP) likely influencing its distribution through seed dispersal [70]. Consequently, certain observable fruit traits and defenses against herbivory, such as spiny trunks and hard epicarp, could be considered anachronic, representing interactions that occurred in the past [70]. The term "anachronic" denotes traits or features that are outdated or no longer relevant in the current context but may have been adaptive or functional in the past [66,70]. While most large mammals are now extinct in the Neotropics following the Late Quaternary Extinction episode, neotropical palms like *Acrocomia spp.* diversified in an ecological environment where mammalian assemblages were abundant in large-bodied species, approximately from 2.6 million to 23,000 BP [66,71,72]. However, our findings suggest only a certain level of connectivity between populations within the two major gene pools. Currently, cattle and humans are regarded as the primary contributors to seed dispersal for this species, highlighting its recent close association with human activities [69,73].

Archaeological evidence encompassing Pre-Columbian civilizations across the neotropics, from Mesoamerica (northern Mexico, Central America) to South America, suggests the cultivation and ritualistic utilization of *Acrocomia aculeata* [18,19]. The presence of *Acrocomia* in these regions indicates a potential influence of Pre-columbian migration on species diversification and offers insights into its domestication process. Such historical records reinforce the relationship between indigenous populations and *Acrocomia*, potentially shaping its genetic diversity and evolutionary trajectory through intentional cultivation and cultural practices. Further investigation is necessary to elucidate the specific historical events and ecological factors shaping the observed genetic structure and center of origin.

Acrocomia totai exhibits a pronounced genetic structuring, showing a clear differentiation between populations from the Brazilian Cerrado, Pantanal, and Chaco and Espinal (Figs 2 and 3). This contrasts with previous studies focused only on neutral loci using SNPs and microsatellites where they observed less genetic structuring among populations [3,74]. This indicates that the populations of *A. totai* are experiencing a recent diversification and ongoing speciation processes. *A. totai* has a subtropical distribution and is adapted to temperate conditions, with selective signatures found in genes associated with fatty acid and triacylglycerol biosynthesis, pathogen resistance and defense, as well as genes specialized in adapting to abiotic and environmental stress (Table 1; S1 and S2 Tables). Pantanal populations exhibit lower levels of admixture compared to other genomic groups within *A. totai*, particularly those distributed in the Brazilian Cerrado. The Cerrado lowlands and ecotones with other local biomes serve as a bridge, including for the South American gene pool of *A. aculeata*. Hybridization between these two species has been reported [3]. Phenotypic differences were also observed in other studies, compared to fruits from the Pantanal, fruits from Cerrado had a higher proportion of epicarp and a lower proportion of kernels [5]. The Pantanal biome, an active sedimentary basin characterized by faults and earthquakes, experiences subsidence and depressions that are highly susceptible to flooding. During the late Pleistocene (14,000–10,000 BP), arid conditions predominated, possibly allowing *A. totai* to recently colonize the biome [75]. The Pantanal lacks endemic tree species, with the majority of terrestrial species being immigrants from the Cerrado [76]. Similar biogeographic patterns are observed in the palm *Copernicia alba* Morong [77]. The domestication process of *A. totai* remains poorly understood, although archaeological records indicate its use for fiber production in Argentina [19]. Human utilization varies among *Acrocomia* species, with *A. totai* being utilized for the yellowish pulp of the fruit for flour production, and the oil derived from the seed, which are the two products of greatest interest [5,13,78].

A. intumescens presented a genetic structure influenced by an allopatric isolation across various phytophysiognomies within Caatinga, rather than by variations in biomes (Figs 2 and 3) [79,80]. Specifically, notable genetic structuring was observed between populations in the North Sertaneja depression and those transitioning from the Borborema highlands to the South Sertaneja depression within the Caatinga biome. The Caatinga exhibits high levels of endemism, with species adapted to survive in its arid climate characterized by drought conditions. The long-term stability of the Caatinga, along with the assembly of ancient plants, has been significantly influenced by aridification processes, while recent vegetation shifts and climate change have driven in situ diversifications. The increased environmental variability has led to the appearance of modern species through Pleistocene/Pliocene (2.6 million BP) ecological specialization [81]. This could elucidate the phylogenomic proximity observed between *A. aculeata* and *A. intumescens*, suggesting a shared common ancestor in the past and probably, *Acrocomia* recently colonized and diversified in the Caatinga [82]. This shared ancestry may be attributed to the dominance of tropical rainforests in the region, which connected the area to the Amazon during the Early Cenozoic period [3,83]. This pattern of endemism and genetic differentiation is shared with other plant species in the Caatinga, including other palms such as *Copernicia prunifera* (Miller) H.E. Moore, legumes (*Coursetia*, *Vatairea*, and *Luetzelburgia* genera), as well as other clades such as *Conopophaga cearae* (Aves. common name: Caatinga gnateater) and lizard species [80,81,84,85]. In *A. intumescens*, the only selective signature found in a gene was associated with stress signaling.

The close relationships observed among *Acrocomia* species may be attributed to the radiation of the Bactridinae subtribe towards the end of the Eocene (23 million BP), when the ancestor of *Acrocomia* diversified [86]. This period of divergence coincided with the terminal Eocene cooling event, characterized by substantial climatic changes that led to accelerated turnover in flora and fauna [87]. This pattern is also evident in the conserved genomic structures and similarity of *Acrocomia aculeata*, *A. totai* and, *A. intumescens* in their plastomes architecture and nuclear gene phylogeny, low pairwise Fst indicating a close relationships [3,83]. The high admixture between populations and the short branch lengths observed in phylogenomic studies, which separate the three species, presents challenges in establishing unambiguous intergeneric relationships within the genus *Acrocomia*. This may suggest a large-scale species-level extinction followed by rapid diversification of surviving lineages.

Correlations between allele frequencies and climatic variables in the LFMM analysis revealed five climatic variables influencing adaptation in the three species: annual mean temperature (BIO1), mean diurnal range (BIO2), isothermality (BIO3), annual precipitation (BIO12), and precipitation of the driest month (BIO14), indicating selection as a driving force of evolutionary adaptation (S2 Fig). Similar patterns were observed in photosynthesis rates and fruit pulp mass development in other *Acrocomia* studies and in the neotropical palm *Mauritia flexuosa* Mart., which is influenced by BIO1, BIO2, and BIO14 [7,88]. Although *Acrocomia* can thrive in a range of climatic conditions from humid to semi-arid ecosystems, they are found exclusively in open areas that are often influenced by human activities. Our results are supported by previous studies that suggest temperature played a significant role in the evolution of the climate niche in Neotropical palm species [9,89].

The sNMF analysis (S2 Fig) showed low differentiation among populations from different biomes in putative SNPs under selection. However, there was evidence of admixture in *Acrocomia totai*, particularly between natural populations from Cerrado in Paraguay, Mato Grosso do Sul (Brazil), Parana (Brazil), and Sao Paulo (Brazil). Biogeographic isolation by barriers is an important driver of genetic differentiation in palm as described by Eiserhardt *et al.:* “The significant effect of spatial distance on clade turnover in American palms can thus be interpreted as evidence for dispersal limitation on evolutionary timescales caused either by barriers or by time” [9]. Even though Eiserharth's results focus on macroevolutionary diversification and phylogenetic turnover by dispersal and niche evolution, they indicate that barrier distance explains more variation in diversity than spatial distance. We observe a similar pattern in our results, where biogeographic barriers are more relevant in shaping genetic structure than biomes proximity and microclimatic variables in *Acrocomia* niches (Fig 3).

Palms are not naturally adapted to withstand extremely high or low temperatures. Interestingly, the evolution of these geographic range restrictions does not appear to be a limiting factor for *Acrocomia*, despite the fact that water supply most likely limits the regional ranges of many neotropical palms [90]. *Acrocomia* appears to be highly adapted to semi-arid ecosystems. The distributions of neotropical palm species are assumed to be impacted by edaphic environments, despite the absence of empirical data. It is known that the natural occurrence in Brazilian populations of *A. aculeata* is associated with eutrophic (high fertility) soils with medium to clayey textures and an average pH of 5.5 [91]. However, further investigation is needed for the Central American gene pool characteristics.

Domestication syndrome in palms is poorly understood [16,92]. Detecting selective sweep regions associated with domestication in *Acrocomia* is crucial. These genes probably have undergone strong positive selection during this process. Selective sweeps are characterized by a reduction in genetic diversity around the selected allele due to the rapid increase in frequency of advantageous alleles favored by human-driven selection [93]. Maybe there is a heterogeneous origin in the domestication of *Acrocomia*. However, the existence of several founder lineages and the link between a mosaic ancestry patterns only will be clear with a sequenced reference genome for the three species. The identification of loci under selection can be greatly enhanced by having access to a reference genome [21]. *Acrocomia* may respond polygenically to environmental variation like other palms [25,88,94], meaning that selection may result in minimal changes in allele frequencies, leading to a poor adaptation signal at a single locus.

Our results demonstrate that genetic structure in populations from different biomes is homogenized within the same biogeographic regions due to gene flow. This also suggests the potential for migration to disperse advantageous alleles among populations sharing the same biogeographic region, leading to allele fixation in genes involved in adaptation to environmental change. This potentially explains *Acrocomia*'s tolerance to seasonality and sheds light on the species' interaction with humans and its domestication process. We suggest investigating the functionality of distinct biological pathways under diverse conditions using a transcriptomic approach for understanding better its domestication syndrome and its potential resilience to climate change [7,89]. Also, conservation strategies can be formulated by identifying genetically distinct populations that may require special attention for conservation.

Genes putatively involved in adaptation and implications for domestication in *Acrocomia*

A high-quality reference genome sequence and annotation at chromosomal level of plant species is essential for genetic research on crop breeding and domestication [20]. *Acrocomia* is considered an incipiently domesticated plant and does not have a reference genome yet, so a population genomics approach using GBS or RAD-Seq can help investigate the evolutionary mechanisms underlying its diversification and variation. Significant genomic alterations have occurred during plant domestication as a result of evolutionary processes such as genetic drift and artificial selection. Studies of artificial selection use genomic techniques to identify signatures that are likely to be subject to selection, with a focus on identifying polymorphisms in trait loci that have been masked by neutral variation in species undergoing a domestication process [21].

Despite lacking a complete reference genome for *Acrocomia*, we were able to analyze genes potentially under selection. To do this, Blast2GO and Blastx use different reference genomes of related palm species to compare the loci where signatures of potential adaptation were located. The most common similarity matches between our GBS tags and annotated sequences were found mostly in homologous genes of *Elaeis guineensis* (African oil palm), *Cocos nucifera* (coconut), and *Phoenix dactylifera* (date palm) (Table 1). For example, phylogenomic studies utilizing chloroplast genome sequences of *A. aculeata* and *E. guineensis* demonstrated that all nodes had a posterior probability of 1.0 (PP = 1.0), indicating a close relationship between these species, eventhough male and female inflorescences are separated in *E. guineensis* while both occur in the same inflorescence in *Acrocomia* spp. [83]. Here, we present some relevant candidate genes that exhibit selection signatures and could be involved in the adaptation processes (and maybe the domestication) of *Acrocomia*.

Some significant selection signatures were identified in genes associated with fatty acid and triacylglycerol biosynthesis pathways in *A. aculeata* (Table 1). For example, loci such as CLocus_25725 correspond to the gene encoding a lipid-binding protein that facilitates lipid transport. CLocus_11291 corresponds to the gene encoding a patatin-like protein 2, which exhibits phospholipase activity in lipid catabolic processes and acylglycerol lipase activity. Additionally, CLocus_32860 was found in the gene encoding an ABC transporter D family member 1, involved in lipid metabolism functions such as the transport of long-chain fatty acids, import of long-chain fatty acids into peroxisomes, and fatty-acyl-CoA transport. Furthermore, CLocus_27100 corresponds to the gene encoding a putative WRKY transcription factor that modulates the content of fatty acids and influences the accumulation of seed oil. Additionally, we found signatures like CLocus_24655 and CLocus_12388 related to genes involved in zinc ion binding processes. In the research led by Couto *et al.*, candidate genes related to oil production traits in *A. aculeata* were identified [95]. The genome-wide association study (GWAS) suggested that candidate genes controlling oil production were associated with metal ion binding and correlated with traits such as fruit oil content, fruit pulp fresh mass, leaf number, and leaf length. In *A. totai*, the locus CLocus_17145 was present in the gene encoding GDSL esterase/lipase, which mobilizes the lipids stored in seeds and plays a major role in seed germination and early seedling establishment [52]. These SNPs could be of agronomic importance as one of the primary objectives of the *Acrocomia* breeding program is to increase oil content for industrial purposes such as biofuels.

These fatty acids and triacylglycerols biosynthesis pathways were also reported in the fine mapping and cross-validation of QTLs linked to fatty acid composition in different varieties of *E. guineensis* made by Ding *et al.* [52]. The Blast results for their QTL included genes and transcription factors linked to diacylglycerol acyltransferase (DGAT1) and long chain acyl-CoA synthetase. The synthesis of triacylglycerol and fatty acids occurs in different compartments within plants. Specifically, new fatty acid synthesis takes place in the plastid (leucoplast), where the acyl carrier protein (ACP) holds the fatty acid chain as it elongates. Then, acyl-ACP thioesterases hydrolyze the acyl-ACPs, releasing non-esterified fatty acids. These fatty acids are then exported to the endoplasmic reticulum, where they assemble to form triacylglycerol [25,96].

Another significant finding was signatures associated in carotenoid biosynthesis pathways (Table 1). In *A. aculeata*, loci such as CLocus_16512 and CLocus_11820 were associated with genes encoding carotenoid cleavage dioxygenase 7 and carotenoid cleavage dioxygenase 8 homolog B, which play roles in carotene catabolic processes and the production of apocarotenoids. These enzymes, known as carotenoid cleavage dioxygenases (CCDs), also influence shoot and branching inhibition and act on various carotenoid substrates like lycopene or zeaxanthin. Additionally, in *A. totai*, the locus CLocus_24114 was found in the gene encoding 9-cis-epoxycarotenoid dioxygenase NCED6, which is involved in regulating plant growth and seed dormancy. Carotenoids are vital for plant growth and development as they serve as precursors for the synthesis of plant hormones such as strigolactones and abscisic acid (ABA) [34,35]. From a nutritional perspective, the high content of carotenoids in crops is an attractive trait, offering benefits such as mitigating vitamin A deficiency through dietary intake of plant-derived carotenoids. For example, the increasing popularity of orange carrots (*Daucus carota* subsp*. sativus*) may be attributed, at least in part, to the numerous studies highlighting the health advantages associated with carotenoids [97].

Additionally, we observed the presence of outlier SNPs in putative resistance genes from different classes, indicating that natural populations under selection possess important genetic resources for crop defense against pathogens (Table 1). For instance, outlier SNPs such as CLocus_27100, CLocus_317, CLocus_22454, CLocus_29621, CLocus_35203, and CLocus_11291 were identified in genes involved in plant defense and disease resistance responses in *A. aculeata*. In *A. totai*, SNPs such as CLocus_37219, CLocus_29376, and CLocus_12178 were associated with genes involved in plant-pathogen recognition, immune priming, and regulation of defense response to fungus, respectively. Currently, there is local evidence of pests affecting both, natural populations and commercial plantations of *A. aculeata.* Examples include *Cyclocephala forsteri* (family Melolonthidae) and the mealybug *Dysmicoccus brevipes* (Hemiptera: Pseudococcidae), particularly in plantations located in Brazil's central region that are now in the production phase [4,98,99]. Phytosanitary

issues with this novel crop would likely arise during the domestication bottleneck in response to genetic diversity and loss of heterozygosity.

We have identified signatures in genes specialized in adapting to abiotic and environmental stress in *A. aculeata*, such as CLocus_317, CLocus_12388, CLocus_23382, CLocus_25856, and CLocus_32860 (Table 1). In *A. totai*, a signature like CLocus_15942 and CLocus_24114 were found in the gene encoding low-affinity sulfate transporter 3, and 9-cis-epoxycarotenoid dioxygenase NCED6, respectively, responsible for the plant's response to drought and salinity stress [58]. Similarly, in *A. intumescens*, the only signature (CLocus_111127) present on a gene was also associated with stress responses. This may help elucidate how *Acrocomia* species can withstand extreme temperatures and droughts. Drought tolerance is a polygenic, complex quantitative trait [100]. Developments in agricultural physiology and genetics have contributed with important insights into drought tolerance in palm species [94,100,101]. Therefore, improving yield under water-deficient conditions is a major objective in plant breeding. Increasing drought tolerance through traditional breeding is a slow process [102]. *Acrocomia*'s inherited characteristics give it the advantage of being adapted to semi-arid, even arid regions like the Brazilian Caatingas. For the majority of non-model species, the mechanisms driving drought tolerance remain poorly understood and vary among species. In comparison to domesticated species, incipiently or less domesticated populations may have a larger range of ecological adaptations [22]. Crop adaptability to perturbed areas depends on its resistance and resilience to environmental stress, which could be crucial for *Acrocomia*'s adaptation in the face of climate change.

Challenges of reduced representation sequencing (GBS/RAD-seq) in crop adaptation studies

Reduced representation sequencing methods in adaptation studies such as genotyping-by-sequencing (GBS) or restriction-site associated DNA sequencing (RAD-seq) employ high-throughput sequencing to generate high genome-wide marker data. The advantages of these techniques is their capacity to develop genotyping assays without requiring prior genomic knowledge or substantial costs without needing a reference genome for perennial non-model crops like *Acrocomia* [20]. GBS serves as a great alternative for investigating unconventional food plants, natural populations, and wild relatives. Additionally, it has been employed to assess diversity and gene flow between crops and their wild relatives, as demonstrated in Brazilian manioc varieties and other crops [23]. Some challenges in adaptation analysis using GBS data include allelic dropout [103], missing targets in resequencing, false positives selection signatures [104] and the management of missing data [105]. However, the identification of false positives may arise from departures from the model's assumptions and covariance with sampling techniques, population structure, and demographic variables [104]. Generally, combining the SNPs identified by various outlier tests is one approach to address these limitations [23,93].

Meanwhile, it is becoming increasingly evident that many crops have complex ancestries, necessitating additional archaeogenomics data [21]. Because of its wide distribution, to gain a better understanding of the domestication process of *Acrocomia* and possible genes targeted by human selection, a more comprehensive examination of variables including the diversity of wild population substructures and the proportional genetic gains of various natural subpopulations is recommended, especially in the Central American genepool. A pangenomic approach is crucial to consider because plant genomes are dynamic, containing a reservoir of genetic diversity that enables adaptation to different biomes [22]. Future crop varieties will likely require a broader range of genes than those provided by single reference genomes. Developing a pangenome of *Acrocomia* could capture the genomic diversity present in the various gene pools observed in our research.

The promising aspects of *Acrocomia* and future perspectives

After exploring various aspects of population genetics, adaptation processes, and potential target genes for domestication in *Acrocomia* species, our study presents a comprehensive understanding of the genetic structure, evolutionary history, and adaptive mechanisms within this genus. Through advanced genomic techniques such as SNP genotyping and adaptive signatures analyses, we explored the relationships between different *Acrocomia* species and their adaptation to

diverse environmental conditions across neotropical biomes. A high degree of diversity was observed in *Acrocomia* species from various neotropical biomes. This variation may have arisen from the crop's natural history, domestication process and its cultivation in diverse ecosystems with varying human preferences. It is plausible that some of the selection signals are associated with desirable agronomic traits, which could be crucial for the breeding of these palms.

Acrocomia spp. emerges as a promising crops due to their multipurpose potential, offering prospects in oil production, biofuels, food, and pharmaceutical industry [106]. Notably, the oil extracted from *A. aculeata* exhibits quality comparable to African palm oil (*E. guineensis*) while requiring lower water resources and demonstrating adaptability to semi-arid to arid environments and degraded ecosystems. Given that approximately 80% of Brazilian grasslands are classified as degraded due to anthropogenic landscape change, intercropping with perennial plants like *Acrocomia* emerges as a promising solution for restaurating and mitigating soil degradation [4,7]. Currently, Brazil is leading its breeding programs through public-private initiatives focusing on *A. aculeata* and *A. totai* [107]. It is projected that experimental and commercial plantations of *A. aculeata* will reach an area of 200,000 hectares in the next decade [108], with a guaranteed market for the oil and biodiesel production. Significant research progress has been made, particularly in seed germination, seedling production, and integrated agricultural systems, although molecular breeding remains necessary [2,4]. Prioritizing the sequencing and annotation of the entire genomes of the three most economically important species in the genus is crucial for future genomic efforts in their domestication process. Addressing challenges such as genotype-environment interaction, biodiversity prospecting for phenotypic breeding ideotypes (plant models), and crop management are necessary. Most research efforts have focused only on the South American gene pool.

Our findings reveal significant genetic structuring within and between species, highlighting the influence of dispersal, biogeographic barriers, and historical ecological interactions on the evolution of *Acrocomia*. We observed distinct genetic signatures associated with adaptation to specific environmental factors, including climatic variables, pathogen resistance, and stress responses. Notably, *Acrocomia* species exhibit potential resilience to climate change, particularly in semi-arid ecosystems. Furthermore, our study sheds light on the potential breeding of *Acrocomia* species, emphasizing the importance of genetic resources and candidate genes associated with desirable agronomic traits such as oil content, carotenoid biosynthesis, and stress tolerance.

These findings significantly advance the field of plant genomics and breeding by providing valuable insights into the genetic mechanisms underlying adaptation in non-model perennial species, particularly within the context of biogeographic and environmental variation. By using advanced genomic tools and gene ontology analyses, this study provides an extensive catalog of loci potentially under selection, which can be used to inform future breeding programs focused on improving resilience and adaptation to changing environmental conditions. While facing challenges inherent to reduced representation sequencing methods, our research underscores the necessity of comprehensive genomic resources, including reference genomes and pangenomic approaches, to fully elucidate the domestication syndrome and facilitate breeding efforts.

Furthermore, the comparative analysis across species not only highlights the role of local adaptation to specific biomes but also reveals species-specific responses to environmental pressures, offering new directions for targeted genomic selection. The inclusion of non-model species in genomic studies opens up new possibilities for incorporating underexplored genetic resources into breeding programs, especially for tropical and subtropical species that are critical to agriculture and sustainable development. This approach facilitates the development of more efficient breeding strategies for crops with enhanced stress tolerance, improved productivity, and greater adaptability to climate change.

Acrocomia is a novel crop offering opportunities for future industries while contributing to sustainable development and biodiversity conservation. By prioritizing collaborative research efforts in private-public programs, genetic conservation, and policy support, different stakeholders can unlocked the potential of genetic diversity and adaptive capacity of *Acrocomia* species to address global challenges, including food security, renewable energy production, and climate resilience in agroecosystems.

Materials and methods

Plant material and DNA isolation

We sampled leaves from natural populations, 78 for *A. aculeata*, 40 for *A. totai*, and 131 for *A. intumescens*, for a total of 249 samples (Table 2). The collections of these samples were registered in the Brazilian National Council for Genetic Patrimony CGEN (numbers A69E071 and A5D139D). The leaves were dehydrated using silica gel and stored in paper bags at −20°C. Following Doyle and Doyle's protocol, we extracted whole genomic DNA from 50 mg leaf samples. Agarose gel electrophoresis (1% w/v) with GelRed stain (Sigma-Aldrich) was used to assess DNA quality and integrity. We quantified and normalized DNA concentrations to 30 ng/µL using the dsDNA BR Assay quantification kit for the Qubit3 fluorometer (Invitrogen).

GBS libraries and SNP discovery

Genomic libraries were prepared following the protocol of genotyping-by-sequencing using two restriction enzymes (ddGBS) as described by Poland *et al*. [109]. The combination of *NsiI* and *MspI* (New England Biolabs) was used for the libraries of *A. aculeata* and *A. totai*, while the combination of *NsiI* and *MseI* (New England Biolabs) was used for *A. intumescens*. Two libraries were prepared mixing *A. aculeata* and *A. totai* samples, each using a 96-plex set of *NsiI* barcode adapters and a common *MspI* adapter. Another two libraries were prepared for *A. intumescens*, each using a 96-plex set of *NsiI* barcode adapters and a common *MseI* adapter. The ddGBS libraries were quantified through RT-PCR on the CFX 384 Touch Real Time PCR (BioRad, USA) equipment using a KAPA Library Quantification kit (KAPA Biosystems, USA, cat. KK4824), and the fragments' profiles were inspected using the Agilent DNA 1000 Kit on a 2100 Bioanalyzer (Agilent Technologies, USA). The libraries of *A. aculeata* and *A. totai* were sequenced on two separate runs in an Illumina NextSeq500 (Illumina, USA), with single-end and 150 bp configurations. The two libraries of *A. intumescens* were sequenced on a single run, but in separate lanes, in an Illumina HiSeq3000 (Illumina, USA), with single-end and 101 bp configurations. The dataset is available in the supporting information (S1 File).

The general sequencing quality and the presence of adapters were assessed with FASTQC [110]. The SNP discovery was performed separately for each species following the *de novo* pipeline of the program Stacks v.1.42 [111] with similar filtering criteria. Due to the presence of adapters, sequences were trimmed to 90 bp for *A. aculeata* and *A. totai*, and 80 pb *A. intumescens*. Trimming, quality control (removal of sequences with uncalled bases and with Phred scores <10), and demultiplexing were performed with the module *process_radtags*. For each sample, loci were assembled using the module *ustacks* (for non-model species that lack a reference genome) considering minimum sequencing depth (-m) of 3, and distance between reads from the same loci (-N) of 2. This module groups alleles into loci using a k-mer search technique. First, a hashing method is applied to organize exactly matching reads into stacks. These stacks are then broken down into k-mers, or subsequences of length k, which are compared across stacks to identify matching alleles [111]. A catalog of loci across samples was built with the module *cstacks*, considering the distance between locus (-n) of 2, and loci of the samples were compared to the catalog using *sstacks*. The loci with lower probabilities (-lnl_lim 10) were discarded using the module *rxstacks*. For the three species, candidate SNPs were identified using the module *populations* considering a minimum depth of 3, minor allele frequency of 0.01, the presence of SNP in at least 75% of the samples in each of 14 (*A. aculeata*), 8 (*A. totai*), or 10 (*A. intumescens*) sampling locations. To avoid explicit linkage only a single SNP was retained per GBS locus. Quality metrics (mean sequencing depth per locus and per sample, percentage of missing data per sample) were assessed with VCFTools [112]. Additionally, samples with more than 50% of missing genotypes were removed from the final data set, resulting in 69 samples for *A.aculeata*, 40 for *A. totai*, and 131 for *A. intumescens*.

Detection of putative signatures of selection

Putative signatures of selection were investigated using complimentary approaches [65,113] either based on significant deviations of F_{ST} estimates among populations (fsthet method), based on principal component analyses (PCA) (pcadapt

Table 2. ***Acrocomia* species samples**. Geographical location and biome of the *Acrocomia* species samples.

Acrocomia aculeata

Country	State	Location	Local Biome	Macro Ecoregion	Lat	Lon
Brazil	Minas Gerais	Cassia	Cerrado	Subtropical Grasslands, Savannas and Shrublands	−20.566412	−46.933644
	Minas Gerais	Ibituruna	Cerrado	Subtropical Grasslands, Savannas and Shrublands	−21.343044	−44.739736
	Minas Gerais	Luz	Cerrado	Subtropical Grasslands, Savannas and Shrublands	−19.773972	−45.864639
	Minas Gerais	Montes Claros	Cerrado	Subtropical Grasslands, Savannas and Shrublands	−16.747219	−43.886300
	Minas Gerais	Patos de Minas	Cerrado	Subtropical Grasslands, Savannas and Shrublands	−18.682009	−46.571756
	Para	Belem	Amazon	Tropical Moist Broadleaf Forest	−1.1446780	−48.146053
	Rio de Janeiro	Guapimirim	Atlantic coastal forest	Subtropical Moist Broadleaf Forest	−22.537222	−42.981944
	Rio de Janeiro	Itaborai	Atlantic coastal forest	Subtropical Moist Broadleaf Forest	−22.714332	−42.810942
	Sao Paulo	Brotas	Cerrado	Subtropical Grasslands, Savannas and Shrublands	−22.276083	−48.118500
	Sao Paulo	Rifania	Cerrado	Subtropical Grasslands, Savannas and Shrublands	−19.986278	−47.508472
	Tocantins	Palmas	Amazon	Tropical Moist Broadleaf Forest	−9.043889	−48.324250
Colombia	Casanare	Aguazul	Llanos	Tropical Dry Broadleaf Forest	5.169456	−72.552262
Costa Rica	Guanacaste	Liberia	Central American moist forest	Tropical Moist Broadleaf Forest	10.603496	−85.429016
	San Jose	San Jose	Central American moist forest	Tropical Moist Broadleaf Forest	9.897481	−84.413358
Mexico	Chiapas	Cacahuatan	Central American moist forest	Tropical Moist Broadleaf Forest	14.980654	−92.172965
	Chiapas	Tuxtla chico	Central American moist forest	Tropical Moist Broadleaf Forest	14.922992	−92.179134
	Quintana Roo	Cancún	Central American moist forest	Tropical Moist Broadleaf Forest	21.153694	−86.842000
	Veracruz	Mangal	Central American moist forest	Tropical Moist Broadleaf Forest	19.002444	−96.159917
Trinidad and Tobago	Tunapuna piarco	Saint George	Caribbean dry forest	Tropical Moist Broadleaf Forest	10.664417	−61.399028

Acrocomia totai

Country	State	Location	Local Biome	Macro Ecoregion	Lat	Lon
Argentina	Formosa	Misión Tacaaglé	Chaco and Espinal	Subtropical Grasslands, Savannas and Shrublands	−24.981556	−58.843472
Paraguay	Itapúa	Bella Vista	Cerrado	Subtropical Grasslands, Savannas and Shrublands	−27.029879	−55.579355
Brazil	Mato Grosso do Sul	Campo Grande	Cerrado	Subtropical Grasslands, Savannas and Shrublands	−20.469056	−54.777361
	Mato Grosso do Sul	Corumba	Pantanal	Flooded Grasslands and Savannas	−19.351261	−57.563631
	Mato Grosso do Sul	Dourados	Cerrado	Subtropical Moist Broadleaf Forest	−22.262750	−54.837889
	Mato Grosso do Sul	Porto Murtinho	Chaco and Espinal	Subtropical Grasslands, Savannas and Shrublands	−21.561294	−57.811203
	Parana	Xambrê	Cerrado	Subtropical Moist Broadleaf Forest	−23.736111	−53.490000
	Sao Paulo	Teodoro Sampaio	Cerrado	Subtropical Moist Broadleaf Forest	−22.536222	−52.183842

(Continued)

Table 2. (Continued)

Acrocomia intumescens						
**Country**	**State**	**Location**	**Local Biome**	**Macro Ecoregion**	**Lat**	**Lon**
Brazil	Ceará	Caririaçu	Caatinga	Tropical Dry Broadleaf Forest	−7.030754	−39.273128
	Ceará	Crato	Caatinga	Tropical Dry Broadleaf Forest	−7.218414	−39.432748
	Ceará	Fortaleza	Atlantic coastal forest	Tropical Dry Broadleaf Forest	−3.881995	−38.502484
	Ceará	Guaramiranga	Atlantic coastal forest	Tropical Dry Broadleaf Forest	−4.261151	−38.928265
	Paraíba	Areia	Caatinga	Tropical Dry Broadleaf Forest	−6.962329	−35.688065
	Paraíba	Campina Grande	Caatinga	Tropical Dry Broadleaf Forest	−7.203068	−35.845533
	Paraíba	Joao Pessoa	Atlantic coastal forest	Tropical Dry Broadleaf Forest	−7.098467	−34.966734
	Paraíba	Mata Limpa	Caatinga	Tropical Dry Broadleaf Forest	−6.938426	−35.712936
	Paraíba	Remigio	Caatinga	Tropical Dry Broadleaf Forest	−6.959532	−35.789893
	Paraíba	Rio Tinto	Atlantic coastal forest	Tropical Dry Broadleaf Forest	−6.823933	−35.060280
	Pernambuco	Recife	Atlantic coastal forest	Tropical Dry Broadleaf Forest	−7.980871	−34.931509

https://doi.org/10.1371/journal.pone.0324340.t001

method), or based on environmental association analyses, which identify association between environmental variables and individual markers.

A classic approach to identify outlier SNPs is the identification of loci with extremely high or low values of F_{ST} (or related statistics) estimated among populations. This method was performed using the FstHet [114], package for R 3.3.1 [115] which implements a model similar to FDist2 [116] to construct a neutral envelope for the distribution of F_{ST}s estimated based on the given data set. The neutral envelope was constructed based on 1,000 bootstraps of the F_{ST}-analogous betahat statistic [117], which considers variations in sampling sizes across populations. In this analysis, the markers below or above the 95% envelope of betahat estimates were considered as outlier SNPs.

Pcadapt [118] was used to identify SNP markers significantly associated with the genetic structure of the data based on a PCA, without assuming any genetic model. This analysis was performed in the pcadapt package for R 3.3.1 [115], retaining the first 3, 4, and 1 K principal components for *A. aculeata*, *A. totai* and *A. intumescens*, respectively (S1 Fig). In this analysis, the SNPs with q-values ≤ 0.1 (corrected p-values for their association with the first K principal components) were considered as outliers.

The Latent Factor Mixed Models (LFMM) analysis [119] was used to assess the correlations of SNP markers with environmental variables obtained in the *WorldClim2* data base [120]. The information recovered from *WorldClim2* refer to 19 bioclimatic variables, of which 11 are primarily related to annual trends of temperature (BIO1 to BIO11) and eight are related to annual trends of precipitation (BIO12 to BIO19). For each species, the variables were extracted for the sampling points, and a Pearson's correlation test was employed to minimize interdependence among them, and only the variables with a correlation coefficient < 0.8 were retained (S2 Fig). A sparse non-negative matrix factorization (sNMF) analysis [121] was performed to estimate the most likely number of genetic clusters for each species to model the covariation of the subjacent genetic structure in LFMM. Both, sNMF and LFMM were performed with the package LEA [122] for R 3.3.1 [115]. For each species, 10 independent repetitions of sNMF simulating from 1 to 10 *K* ancestral groups were performed with 200,000 iterations. The most probable number of $K = 2$ for *A. aculeata* and *A. intumescens*, and $K = 4$ for *A. totai* were estimated according to the cross-entropy estimates of the algorithm (S3 Fig). These numbers were used in subsequent LFMM analyses, which were performed based on 10 repetitions of 50,000 burn-in followed by 100,000 iterations of the algorithm for each species. The generated p-values were corrected considering a false discovery rate (FDR) of 0.1 as threshold for the identification of SNPs significantly associated with the environmental variables.

SNPs were declared as putatively under selection if identified as outliers (or significantly associated to environmental variables) by at least two of the three methods described above. Blast2GO (BioBam, Spain) [123] was used to assess similarities between the GBS tags with outlier SNPs and proteins with described putative functional annotations. The similarity between GBS tags in which outlier SNPs were identified and proteins deposited in GenBank was evaluated using blastx with default configurations but restricting the search to Viridiplantae data. Sequences with blastx hits were then screened against the Pfam database to search for protein domains and their associated Gene Ontology (GO) annotations. The putative annotations were summarized based on the GO terms and visually represented with bar plots using the on-line tool WEGO (Web Gene Ontology Annotation Plotting, China) (http://wego.genomics.cn/).

Supporting information

S1 Fig. Principal component analysis (PCA) performed for outlier detection using pcadapt for *Acrocomia aculeata*, *A. totai* and *A. intumescens*. Top row: scree plots of the explained variance (y-axis) retained in each principal component (PC) (x-axis). The number of retained PCs was chosen as the first inflection point (Cattell's rule), where the amount of genetic variation added by successive PCs reaches a plateau. Bottom row: the associated scatter plots of the first two PCs.
(TIF)

S2 Fig. Bioclimatic variables recovered from WorldClim2 and extracted for the sampling points of *Acrocomia aculeata*, *A. totai* and *A. intumescens*. Groups of intercorrelated variables highlighted within red boxes (Pearson's correlation coefficients > 0.8).
(TIF)

S3 Fig. Sparse non-negative matrix factorization (sNMF) performed for outlier detection using Latent Factor Mixed Models (LFMM) for *Acrocomia aculeata*, *A. totai* and *A. intumescens*. Left: plot of the cross-entropy estimates for each number of simulated ancestral populations. Right: Bar plots representing the sNMF ancestry coefficients across samples from different biogeographic group in each species. Each sample is represented by a bar and different shade colors represent their associated ancestry proportion from distinct genetic groups. Acronyms follow Table 2: CMF Central American Moist Forest; LLA Llanos; CDF Caribbean Dry Forest; ACF Atlantic Coastal Forest; CAA Caatinga; AMA Amazon; CER Cerrado; PAN Pantanal; CHE Chaco and Espinal.
(TIF)

S1 Table. List of loci identified as outliers for each species, based on Pcadapt, FstHet and LFMM. For Pcadapt, "PC" refers to the principal component associated to the outlier locus. For FstHet, estimates of total genetic diversity (Ht) and genetic divergence (Fst) are presented. For LFMM, values within cells are the p-values estimated for the association between loci and the given bioclimatic variable (Excel spreadsheet).
(XLSX)

S2 Table. Summary of Blast2GO analysis. Information of SNP loci putatively under selection and their associated GBS tags are followed by the first significant blastx hit (including protein name, the species for which it was described, and GenBank accession number), and putative annotations recovered as Gene Ontology (GO) terms. E-value = expected probability of spurious hits (Excel spreadsheet).
(XLSX)

S1 File. Morales-Marroquín, Jonathan Andre (2024). SNP genotype data set in variant calling format (vcf) for *Acrocomia* species.
(ZIP)

Author contributions

Conceptualization: Brenda Gabriela Díaz-Hernández, Suelen Alves Vianna, Carlos A. Colombo, Maria Imaculada Zucchi.

Data curation: Jonathan A. Morales-Marroquín, Alessandro Alves-Pereira, Brenda Gabriela Díaz-Hernández.

Formal analysis: Jonathan A. Morales-Marroquín, Alessandro Alves-Pereira, Carlos Eduardo de Araújo Batista, Maria Imaculada Zucchi.

Funding acquisition: Carlos A. Colombo, José Baldin Pinheiro, Maria Imaculada Zucchi.

Investigation: Jonathan A. Morales-Marroquín, Alessandro Alves-Pereira, Brenda Gabriela Díaz-Hernández, Suelen Alves Vianna, Carlos Eduardo de Araújo Batista, Carlos A. Colombo, Maria Imaculada Zucchi.

Methodology: Alessandro Alves-Pereira, Brenda Gabriela Díaz-Hernández, Carlos Eduardo de Araújo Batista.

Project administration: Jonathan A. Morales-Marroquín, Carlos A. Colombo, Maria Imaculada Zucchi.

Resources: Carlos A. Colombo, José Baldin Pinheiro, Maria Imaculada Zucchi.

Software: Alessandro Alves-Pereira.

Supervision: Carlos A. Colombo, José Baldin Pinheiro, Maria Imaculada Zucchi.

Validation: Alessandro Alves-Pereira, Brenda Gabriela Díaz-Hernández, Suelen Alves Vianna, Carlos A. Colombo, Maria Imaculada Zucchi.

Writing – original draft: Jonathan A. Morales-Marroquín.

Writing – review & editing: Jonathan A. Morales-Marroquín, Alessandro Alves-Pereira, Brenda Gabriela Díaz-Hernández, Suelen Alves Vianna, Carlos Eduardo de Araújo Batista, Carlos A. Colombo, José Baldin Pinheiro, Maria Imaculada Zucchi.

References

1. de Lima NE, Carvalho AA, Meerow AW, Manfrin MH. A review of the palm genus Acrocomia: Neotropical green gold. Org Divers Evol. 2018;18(2):151–61. https://doi.org/10.1007/s13127-018-0362-x
2. Vargas-Carpintero R, Hilger T, Mössinger J, Souza RF, Barroso Armas JC, Tiede K, et al. Acrocomia spp.: neglected crop, ballyhooed multipurpose palm or fit for the bioeconomy? A review. Agron Sustain Dev. 2021;41(6):1–35. https://doi.org/10.1007/s13593-021-00729-5
3. Díaz BG, Zucchi MI, Alves-Pereira A, de Almeida CP, Moraes ACL, Vianna SA, et al. Genome-wide SNP analysis to assess the genetic population structure and diversity of Acrocomia species. PLoS One. 2021;16(7):e0241025. https://doi.org/10.1371/journal.pone.0241025 PMID: 34283830
4. Colombo CA, Chorfi Berton LH, Diaz BG, Ferrari RA. Macauba: a promising tropical palm for the production of vegetable oil. OCL. 2018.
5. Ciconini G, Favaro SP, Roscoe R, Miranda CHB, Tapeti CF, Miyahira MAM, et al. Biometry and oil contents of Acrocomia aculeata fruits from the Cerrados and Pantanal biomes in Mato Grosso do Sul, Brazil. Ind Crops Prod. 2013;45:208–14.
6. Vianna SA, Carmelo-Guerreiro SM, Noblick LR, Colombo CA. Leaf anatomy of Acrocomia (Arecaceae): an additional contribution to the taxonomic resolution of a genus with great economic potential. Plant Syst Evol. 2017;303:233–48.
7. Resende RT, Kuki KN, Corrêa TR, Zaidan ÚR, Mota PHS, Telles LAA, et al. Data-based agroecological zoning of Acrocomia aculeata: GIS modeling and ecophysiological aspects into a Brazilian representative occurrence area. Ind Crops Prod. 2020;154:112749.
8. Alfaro-Solís JD, Montoya-Arroyo A, Jiménez VM, Arnáez-Serrano E, Pérez J, Vetter W, et al. Acrocomia aculeata fruits from three regions in Costa Rica: an assessment of biometric parameters, oil content and oil fatty acid composition to evaluate industrial potential. Agroforest Syst. 2020;94(5):1913–27. https://doi.org/10.1007/s10457-020-00511-8
9. Eiserhardt WL, Svenning J-C, Baker WJ, Couvreur TLP, Balslev H. Dispersal and niche evolution jointly shape the geographic turnover of phylogenetic clades across continents. Sci Rep. 2013;3:1164. https://doi.org/10.1038/srep01164 PMID: 23383367
10. Dinerstein E, Olson D, Joshi A, Vynne C, Burgess ND, Wikramanayake E, et al. An Ecoregion-Based Approach to Protecting Half the Terrestrial Realm. Bioscience. 2017;67(6):534–45. https://doi.org/10.1093/biosci/bix014 PMID: 28608869
11. Lorenzi H, Noblick L, Kahn F, Ferreira E. Flora Brasileira Arecaceae (Palmeiras). Inst. Plant. Estud. Da Flora Ltda. Nova Odessa: Instituto Plantarum; 2010.
12. Freitas CG, Bacon CD, Souza-Neto AC, Collevatti RG. Adjacency and Area Explain Species Bioregional Shifts in Neotropical Palms. Front Plant Sci. 2019;10:55. https://doi.org/10.3389/fpls.2019.00055 PMID: 30804955

13. Vianna SA, Domenech HLM, da Silva RH, Colombo CA, Pott A. Morphological characterization and productivity estimates of Acrocomia totai Mart. (Arecaceae) – a sustainable alternative of extractivism and cultivation. Rev Bras Frutic [Internet]. 2021 [cited 2024 Mar 18];43:e-730. Available from: https://www.scielo.br/j/rbf/a/LfmCRkWjDxHRvHMRgbwyHgN/
14. Nascimento ADPD, Soares LAL, Stragevitch L, Danielski L. Extraction of Acrocomia intumescens Drude oil with supercritical carbon dioxide: Process modeling and comparison with organic solvent extractions. J Supercrit Fluids. 2016;111:1–7.
15. Morcote-Ríos G, Bernal R. Remains of palms (Palmae) at archaeological sites in the New World: A review. Bot Rev. 2001;67(3):309–50. https://doi.org/10.1007/bf02858098
16. Clement CR. 1492 and the loss of amazonian crop genetic resources. I. The relation between domestication and human population decline. Econ Bot. 1999;53(2):188–202. https://doi.org/10.1007/bf02866498
17. Clement CR. 1492 and the loss of amazonian crop genetic resources. ii. crop Biogeography at contact. Econ Bot. 1999;53(2):203–16. https://doi.org/10.1007/bf02866499
18. Ramírez-Núñez C, Cyphers A, Parrot JF, Höfle B. Multidirectional interpolation of lidar data from southern Veracruz, Mexico: implications for early olmec subsistence. Anc Mesoamerica. 2019.
19. Rodríguez MF, Aschero CA. Acrocomia chunta (Arecaceae) raw material for cord making in the Argentinean Puna. J Archaeol Sci. 2005;32:1534–42.
20. Schreiber M, Stein N, Mascher M. Genomic approaches for studying crop evolution. Genome Biol. 2018;19(1):140. https://doi.org/10.1186/s13059-018-1528-8 PMID: 30241487
21. Huang X, Huang S, Han B, Li J. The integrated genomics of crop domestication and breeding. Cell. 2022;185(15):2828–39. https://doi.org/10.1016/j.cell.2022.04.036 PMID: 35643084
22. Petereit J, Bayer PE, Thomas WJW, Tay Fernandez CG, Amas J, Zhang Y, et al. Pangenomics and Crop Genome Adaptation in a Changing Climate. Plants [Internet]. 2022 [cited 2024 Mar 8];11. Available from: https://pmc/articles/PMC9370458/
23. Alves-Pereira A, Zucchi MI, Clement CR, Viana JPG, Pinheiro JB, Veasey EA, et al. Selective signatures and high genome-wide diversity in traditional Brazilian manioc (*Manihot esculenta* Crantz) varieties. Sci Reports [Internet]. 2022 [cited 2024 Feb 22];12:1–14. Available from: https://www.nature.com/articles/s41598-022-05160-8
24. Kang J, Park J, Choi H, Burla B, Kretzschmar T, Lee Y, et al. Plant ABC Transporters. Arabidopsis Book. 2011;9:e0153. https://doi.org/10.1199/tab.0153 PMID: 22303277
25. Ting N-C, Yaakub Z, Kamaruddin K, Mayes S, Massawe F, Sambanthamurthi R, et al. Fine-mapping and cross-validation of QTLs linked to fatty acid composition in multiple independent interspecific crosses of oil palm. BMC Genomics. 2016;17:289. https://doi.org/10.1186/s12864-016-2607-4 PMID: 27079197
26. Guo Z-H, Lung S-C, Fadhli Hamdan M, Chye M-L. Interactions between plant lipid-binding proteins and their ligands. Prog Lipid Res. 2022;86:101156. https://doi.org/10.1016/j.plipres.2022.101156 PMID: 35066006
27. La Camera S, Balagué C, Göbel C, Geoffroy P, Legrand M, Feussner I, et al. The Arabidopsis patatin-like protein 2 (PLP2) plays an essential role in cell death execution and differentially affects biosynthesis of oxylipins and resistance to pathogens. Mol Plant Microbe Interact. 2009;22(4):469–81. https://doi.org/10.1094/MPMI-22-4-0469 PMID: 19271961
28. Barkan A, Small I. Pentatricopeptide repeat proteins in plants. Annu Rev Plant Biol [Internet]. 2014 [cited 2024 Mar 10];65:415–42. Available from: https://pubmed.ncbi.nlm.nih.gov/24471833/
29. Habsyah TS, Basyuni M, Siregar LAM, Afandi D, Syahputra I. Distribution of polyprenol and dolichol in oil palm genotype (*Elaeis guineensis*) involving lipase activity. Biodiversitas J Biol Divers [Internet]. 2021 [cited 2022 Aug 16];22:830–7. Available from: https://smujo.id/biodiv/article/view/7415
30. Van Gelder K, Virta LKA, Easlick J, Prudhomme N, McAlister JA, Geddes-McAlister J, et al. A central role for polyprenol reductase in plant dolichol biosynthesis. Plant Sci [Internet]. 2021 [cited 2022 Sep 5];303. Available from: https://pubmed.ncbi.nlm.nih.gov/33487357/
31. Cheng Y, Jalalahammed G, Yu J, Yao Z, Ruan M, Ye Q, et al. Putative WRKYs associated with regulation of fruit ripening revealed by detailed expression analysis of the WRKY gene family in pepper. Sci Reports [Internet]. 2016 [cited 2022 Aug 16];6:1–11. Available from: https://www.nature.com/articles/srep39000
32. Khoso MA, Hussain A, Ritonga FN, Ali Q, Channa MM, Alshegaihi RM, et al. WRKY transcription factors (TFs): Molecular switches to regulate drought, temperature, and salinity stresses in plants. Front Plant Sci. 2022;13:1039329. https://doi.org/10.3389/fpls.2022.1039329 PMID: 36426143
33. Booker J, Auldridge M, Wills S, McCarty D, Klee H, Leyser O. MAX3/CCD7 is a carotenoid cleavage dioxygenase required for the synthesis of a novel plant signaling molecule. Curr Biol. 2004;14(14):1232–8. https://doi.org/10.1016/j.cub.2004.06.061 PMID: 15268852
34. Mishra S, Upadhyay S, Shukla RK. The Role of Strigolactones and Their Potential Cross-talk under Hostile Ecological Conditions in Plants. Front Physiol. 2017;7:691. https://doi.org/10.3389/fphys.2016.00691 PMID: 28119634
35. Saeed W, Naseem S, Ali Z. Strigolactones biosynthesis and their role in abiotic stress resilience in plants: A critical review. Front Plant Sci. 2017.
36. Yao Y, Jia L, Cheng Y, Ruan M, Ye Q, Wang R, et al. Evolutionary Origin of the Carotenoid Cleavage Oxygenase Family in Plants and Expression of Pepper Genes in Response to Abiotic Stresses. Front Plant Sci. 2022;12:792832. https://doi.org/10.3389/fpls.2021.792832 PMID: 35126418

37. Liu J, Novero M, Charnikhova T, Ferrandino A, Schubert A, Ruyter-Spira C, et al. Carotenoid cleavage dioxygenase 7 modulates plant growth, reproduction, senescence, and determinate nodulation in the model legume *Lotus japonicus*. J Exp Bot [Internet]. 2013 [cited 2022 Aug 16];64:1967–81. Available from: https://academic.oup.com/jxb/article/64/7/1967/581146

38. Sorefan K, Booker J, Haurogné K, Goussot M, Bainbridge K, Foo E, et al. MAX4 and RMS1 are orthologous dioxygenase-like genes that regulate shoot branching in *Arabidopsis* and pea. Genes Dev [Internet]. 2003 [cited 2022 Sep 6];17:1469–74. Available from: https://pubmed.ncbi.nlm.nih.gov/12815068/

39. Schwartz SH, Qin X, Loewen MC. The biochemical characterization of two carotenoid cleavage enzymes from *Arabidopsis* indicates that a carotenoid-derived compound inhibits lateral branching. J Biol Chem [Internet]. 2004 [cited 2022 Sep 6];279:46940–5. Available from: https://pubmed.ncbi.nlm.nih.gov/15342640/

40. Lee BH, Wynn AN, Franks RG, Hwang Y, Lim J, Kim JH. The Arabidopsis thaliana GRF-INTERACTING FACTOR gene family plays an essential role in control of male and female reproductive development. Dev Biol. 2014;386(1):12–24. https://doi.org/10.1016/j.ydbio.2013.12.009 PMID: 24355747

41. Lu Y, Meng Y, Zeng J, Luo Y, Feng Z, Bian L, et al. Coordination between GROWTH-REGULATING FACTOR1 and GRF-INTERACTING FACTOR1 plays a key role in regulating leaf growth in rice. BMC Plant Biol. 2020;20(1):200. https://doi.org/10.1186/s12870-020-02417-0 PMID: 32384927

42. Saiga S, Möller B, Watanabe-Taneda A, Abe M, Weijers D, Komeda Y. Control of embryonic meristem initiation in *Arabidopsis* by PHD-finger protein complexes. Development [Internet]. 2012 [cited 2022 Sep 6];139:1391–8. Available from: https://pubmed.ncbi.nlm.nih.gov/22378640/

43. Lin TF, Saiga S, Abe M, Laux T. OBE3 and WUS Interaction in Shoot Meristem Stem Cell Regulation. PLoS One [Internet]. 2016 [cited 2022 Sep 6];11. Available from: https://pubmed.ncbi.nlm.nih.gov/27196372/

44. Backer R, Naidoo S, van den Berg N. The NONEXPRESSOR OF PATHOGENESIS-RELATED GENES 1 (NPR1) and Related Family: Mechanistic Insights in Plant Disease Resistance. Front Plant Sci. 2019;10:102. https://doi.org/10.3389/fpls.2019.00102 PMID: 30815005

45. Perrot T, Pauly M, Ramírez V. Emerging Roles of β-Glucanases in Plant Development and Adaptative Responses. Plants (Basel). 2022;11(9):1119. https://doi.org/10.3390/plants11091119 PMID: 35567119

46. Shimura K, Okada A, Okada K, Jikumaru Y, Ko KW, Toyomasu T, et al. Identification of a biosynthetic gene cluster in rice for momilactones. J Biol Chem [Internet]. 2007 [cited 2022 Sep 6];282:34013–8. Available from: https://pubmed.ncbi.nlm.nih.gov/17872948/

47. Zhang J, Peters RJ. Why are momilactones always associated with biosynthetic gene clusters in plants? Proc Natl Acad Sci U S A. 2020;117(25):13867–9. https://doi.org/10.1073/pnas.2007934117 PMID: 32487731

48. Figueiredo A, Monteiro F, Sebastiana M. Subtilisin-like proteases in plant-pathogen recognition and immune priming: a perspective. Front Plant Sci. 2014;5:739. https://doi.org/10.3389/fpls.2014.00739 PMID: 25566306

49. Zhang L, Jim enez-G omez JM. Functional analysis of FRIGIDA using naturally occurring variation in Arabidopsis thaliana. 2020.

50. Rupwate SD, Rajasekharan R. Plant phosphoinositide-specific phospholipase C: an insight. Plant Signal Behav. 2012;7(10):1281–3. https://doi.org/10.4161/psb.21436 PMID: 22902702

51. Shen G, Sun W, Chen Z, Shi L, Hong J, Shi J. Plant GDSL Esterases/Lipases: Evolutionary, Physiological and Molecular Functions in Plant Development. Plants (Basel). 2022;11(4):468. https://doi.org/10.3390/plants11040468 PMID: 35214802

52. Ding Y, Xing L, Xu J, Jiang T, Tang X, Wang Y, et al. Genome-wide exploration of the GDSL-type esterase/lipase gene family in rapeseed reveals several BnGELP proteins active during early seedling development. Front Plant Sci. 2023;14:1139972. https://doi.org/10.3389/fpls.2023.1139972 PMID: 37008509

53. Li J, Galla A, Avila CA, Flattmann K, Vaughn K, Goggin FL. Fatty Acid Desaturases in the Chloroplast and Endoplasmic Reticulum Promote Susceptibility to the Green Peach Aphid Myzus persicae in Arabidopsis thaliana. Mol Plant Microbe Interact. 2021;34(6):691–702. https://doi.org/10.1094/MPMI-12-20-0345-R PMID: 33596108

54. Jin H, Axtell MJ, Dahlbeck D, Ekwenna O, Zhang S, Staskawicz B, et al. NPK1, an MEKK1-like mitogen-activated protein kinase kinase kinase, regulates innate immunity and development in plants. Dev Cell. 2002;3(2):291–7. https://doi.org/10.1016/s1534-5807(02)00205-8 PMID: 12194859

55. Noack LC, Bayle V, Armengot L, Rozier F, Mamode-Cassim A, Stevens FD, et al. A nanodomain-anchored scaffolding complex is required for the function and localization of phosphatidylinositol 4-kinase alpha in plants. The Plant Cell. 2021;34(1):302–32. https://doi.org/10.1093/plcell/koab135

56. Takáč T, Novák D, Šamaj J. Recent Advances in the Cellular and Developmental Biology of Phospholipases in Plants. Front Plant Sci. 2019;10:362. https://doi.org/10.3389/fpls.2019.00362 PMID: 31024579

57. Wu G, Liu S, Zhao Y, Wang W, Kong Z, Tang D. ENHANCED DISEASE RESISTANCE4 associates with CLATHRIN HEAVY CHAIN2 and modulates plant immunity by regulating relocation of EDR1 in Arabidopsis. Plant Cell. 2015;27(3):857–73. https://doi.org/10.1105/tpc.114.134668 PMID: 25747881

58. Gallardo K, Courty P-E, Le Signor C, Wipf D, Vernoud V. Sulfate transporters in the plant's response to drought and salinity: regulation and possible functions. Front Plant Sci. 2014;5:580. https://doi.org/10.3389/fpls.2014.00580 PMID: 25400648

59. Martínez-Andújar C, Ordiz MI, Huang Z, Nonogaki M, Beachy RN, Nonogaki H. Induction of 9-cis-epoxycarotenoid dioxygenase in Arabidopsis thaliana seeds enhances seed dormancy. Proc Natl Acad Sci U S A. 2011;108(41):17225–9. https://doi.org/10.1073/pnas.1112151108 PMID: 21969557

60. Huang Y, Guo Y, Liu Y, Zhang F, Wang Z, Wang H, et al. 9-cis-Epoxycarotenoid Dioxygenase 3 Regulates Plant Growth and Enhances Multi-Abiotic Stress Tolerance in Rice. Front Plant Sci. 2018;9:162. https://doi.org/10.3389/fpls.2018.00162 PMID: 29559982

61. He R, Zhuang Y, Cai Y, Agüero CB, Liu S, Wu J, et al. Overexpression of 9-cis-Epoxycarotenoid Dioxygenase Cisgene in Grapevine Increases Drought Tolerance and Results in Pleiotropic Effects. Front Plant Sci. 2018;9:970. https://doi.org/10.3389/fpls.2018.00970 PMID: 30123225
62. Xuan TD, Minh TN, Anh LH, Khanh TD. Allelopathic momilactones A and B are implied in rice drought and salinity tolerance, not weed resistance. Agron Sustain Dev. 2016;36(3):1–8. https://doi.org/10.1007/s13593-016-0383-9
63. Kato-Noguchi H. Defensive Molecules Momilactones A and B: Function, Biosynthesis, Induction and Occurrence. Toxins (Basel). 2023;15(4):241. https://doi.org/10.3390/toxins15040241 PMID: 37104180
64. Fuchs S, Grill E, Meskiene I, Schweighofer A. Type 2C protein phosphatases in plants. FEBS J. 2013;280(2):681–93. https://doi.org/10.1111/j.1742-4658.2012.08670.x PMID: 22726910
65. Lotterhos KE, Whitlock MC. The relative power of genome scans to detect local adaptation depends on sampling design and statistical method. Mol Ecol. 2015;24(5):1031–46. https://doi.org/10.1111/mec.13100 PMID: 25648189
66. Nascimento LFd, Guimarães PR, Onstein RE, Kissling WD, Pires MM. Associated evolution of fruit size, fruit colour and spines in Neotropical palms. J Evol Biol. 2020;33(6):858–68. https://doi.org/10.1111/jeb.13619 PMID: 32198956
67. Bitocchi E, Nanni L, Bellucci E, Rossi M, Giardini A, Zeuli PS, et al. Mesoamerican origin of the common bean (Phaseolus vulgaris L.) is revealed by sequence data. Proc Natl Acad Sci U S A. 2012;109(14):E788-96. https://doi.org/10.1073/pnas.1108973109 PMID: 22393017
68. Bedoya CA, Dreisigacker S, Hearne S, Franco J, Mir C, Prasanna BM, et al. Genetic diversity and population structure of native maize populations in Latin America and the Caribbean. PLoS One. 2017;12(4):e0173488. https://doi.org/10.1371/journal.pone.0173488 PMID: 28403177
69. Göldel B, Araujo AC, Kissling WD, Svenning J-C. Impacts of large herbivores on spinescence and abundance of palms in the Pantanal, Brazil. Bot J Linn Soc. 2016;182(2):465–79. https://doi.org/10.1111/boj.12420
70. Janzen DH, Martin PS. Neotropical anachronisms: the fruits the gomphotheres ate. Science. 1982;215(4528):19–27. https://doi.org/10.1126/science.215.4528.19 PMID: 17790450
71. Baker WJ, Couvreur TLP. Global biogeography and diversification of palms sheds light on the evolution of tropical lineages. I. Historical biogeography. J Biogeograph. 2012;40(2):274–85. https://doi.org/10.1111/j.1365-2699.2012.02795.x
72. Villavicencio NA, Lindsey EL, Martin FM, Borrero LA, Moreno PI, Marshall CR, et al. Combination of humans, climate, and vegetation change triggered Late Quaternary megafauna extinction in the Última Esperanza region, southern Patagonia, Chile. Ecography. 2015;39(2):125–40. https://doi.org/10.1111/ecog.01606
73. Lentz DL. Acrocomia mexicana: Palm of the ancient Mesoamericans. J Ethnobiol. 1990;10:183–94.
74. de Lima NE, Meerow AW, Manfrin MH. Genetic structure of two Acrocomia ecotypes (Arecaceae) across Brazilian savannas and seasonally dry forests. Tree Genetics & Genomes. 2020;16(4):1–12. https://doi.org/10.1007/s11295-020-01446-y
75. Assine ML, Merino ER, Pupim FN, Warren LV, Guerreiro RL, McGlue MM. Geology and geomorphology of the pantanal basin. Handb Environ Chem [Internet]. 2016 [cited 2024 Mar 18];37:23–50. Available from: https://link.springer.com/chapter/10.1007/698_2015_349
76. Junk WJ, da Cunha CN, Wantzen KM, Petermann P, Strüssmann C, Marques MI, et al. Biodiversity and its conservation in the Pantanal of Mato Grosso, Brazil. Aquat Sci. 2006;68(3):278–309. https://doi.org/10.1007/s00027-006-0851-4
77. Costa MF, Marroquín JAM, de Araújo Batista CE, Alves-Pereira A, de Almeida Vieira F, Zucchi MI. Population Genomics and Conservation of Copernicia alba: a Non-Timber Palm of Economic Importance in the Brazilian Chaco. Plant Mol Biol Rep. 2023;42(2):235–45. https://doi.org/10.1007/s11105-023-01419-0
78. Sanjinez-Argandoña EJ, Chuba CAM. Caracterização biométrica, física e química de frutos da palmeira bocaiuva *Acrocomia aculeata* (Jacq) Lodd. Rev Bras Frutic [Internet]. 2011 [cited 2024 Mar 22];33:1023–8. Available from: https://www.scielo.br/j/rbf/a/gqQ7Dtjfd5y3RvkjYrhK8Qw/
79. da Silva JMC, Barbosa LCF, Leal IR, Tabarelli M. The Caatinga: Understanding the challenges. Caatinga Larg Trop Dry For Reg South Am [Internet]. 2018 [cited 2024 Mar 13];3–19. Available from: https://link.springer.com/chapter/10.1007/978-3-319-68339-3_1
80. Uchôa LR, Delfim FR, Mesquita DO, Colli GR, Garda AA, Guedes TB. Lizards (Reptilia: Squamata) from the Caatinga, northeastern Brazil: Detailed and updated overview. Vertebr Zool [Internet]. 2022 [cited 2024 Mar 13];72:599–659. Available from: https://vertebrate-zoology.arphahub.com/article/78828/
81. Fernandes MF, Cardoso D, Pennington RT, de Queiroz LP. The Origins and Historical Assembly of the Brazilian Caatinga Seasonally Dry Tropical Forests. Front Ecol Evol. 2022;10:723286.
82. Freitas C, Brum FT, Cássia-Silva C, Maracahipes L, Carlucci MB, Collevatti RG, et al. Incongruent spatial distribution of taxonomic, phylogenetic, and functional diversity in neotropical cocosoid palms. Front For Glob Chang. 2021;4:739468.
83. Francisconi AF, Marroquín JAM, Cauz-Santos LA, van den Berg C, Martins KKM, Costa MF, et al. Complete chloroplast genomes of six neotropical palm species, structural comparison, and evolutionary dynamic patterns. Sci Reports [Internet]. 2023 [cited 2023 Nov 30];13:1–15. Available from: https://www.nature.com/articles/s41598-023-44631-4
84. Costa MF, Morales-Marroquín JA, de Araújo Batista CE, Alves-Pereira A, de Almeida Vieira F, Zucchi MI. Population genomics of the neotropical palm Copernicia prunifera (Miller) H. E. Moore: Implications for conservation. PLoS One. 2022;17(11):e0276408. https://doi.org/10.1371/journal.pone.0276408 PMID: 36327224
85. Batalha-Filho H, Barreto SB, Silveira MHB, Miyaki CY, Afonso S, Ferrand N, et al. Disentangling the contemporary and historical effects of landscape on the population genomic variation of two bird species restricted to the highland forest enclaves of northeastern Brazil. Heredity (Edinb). 2024;132(2):77–88. https://doi.org/10.1038/s41437-023-00662-1 PMID: 37985738

86. Baker WJ, Couvreur TLP. Global biogeography and diversification of palms sheds light on the evolution of tropical lineages. II. Diversification history and origin of regional assemblages. J Biogeograph. 2012;40(2):286–98. https://doi.org/10.1111/j.1365-2699.2012.02794.x
87. Hoorn C, Wesselingh FP, ter Steege H, Bermudez MA, Mora A, Sevink J, et al. Amazonia through time: Andean uplift, climate change, landscape evolution, and biodiversity. Science. 2010;330(6006):927–31. https://doi.org/10.1126/science.1194585 PMID: 21071659
88. Melo WA, Vieira LD, Novaes E, Bacon CD, Collevatti RG. Selective Sweeps Lead to Evolutionary Success in an Amazonian Hyperdominant Palm. Front Genet [Internet]. 2020 [cited 2024 Mar 5];11. Available from: https://pmc/articles/PMC7786001/
89. Walther GR, Gritti ES, Berger S, Hickler T, Tang Z, Sykes MT. Palms tracking climate change. Glob Ecol Biogeogr. 2007;16:801–9.
90. Blach-Overgaard A, Svenning J, Dransfield J, Greve M, Balslev H, et al. Determinants of palm species distributions across Africa: the relative roles of climate, non-climatic environmental factors, and spatial constraints. Ecography. 2010;33(2):380–91. https://doi.org/10.1111/j.1600-0587.2010.06273.x
91. Motoike SY, Carvalho M, Pimentel LD, Kuki KN, Paes JMV, Dias HCT, et al. A cultura da macaúba: implantação e manejo de cultivos racionais. 1st ed. Viçosa, Minas Gerais, Brazil: Universidade Federal de Viçosa; 2013.
92. Levis C, Flores BM, Moreira PA, Luize BG, Alves RP, Franco-Moraes J, et al. How people domesticated Amazonian forests. Front Ecol Evol. 2018;5:299700.
93. Pankin A, Altmüller J, Becker C, von Korff M. Targeted resequencing reveals genomic signatures of barley domestication. New Phytol. 2018;218(3):1247–59. https://doi.org/10.1111/nph.15077 PMID: 29528492
94. Salgado FF, da Silva TLC, Vieira LR, Silva VNB, Leão AP, Costa MM do C, et al. The early response of oil palm (Elaeis guineensis Jacq.) plants to water deprivation: Expression analysis of miRNAs and their putative target genes, and similarities with the response to salinity stress. Front Plant Sci. 2022;13:970113. https://doi.org/10.3389/fpls.2022.970113 PMID: 36212369
95. Couto EGdO, Morales-Marroquín J, Alves-Pereira A, Fernandes SB, Colombo CA, Filho JA de A, et al. Genome-Wide Association Insights into the Genomic Regions Controlling Oil Production Traits in Acrocomia aculeata (neotropical native palm). bioRxiv [Internet]. 2024 [cited 2024 Feb 22];2024.01.17.576138. Available from: https://www.biorxiv.org/content/10.1101/2024.01.17.576138v1
96. Chapman KD, Ohlrogge JB. Compartmentation of triacylglycerol accumulation in plants. J Biol Chem [Internet]. 2012 [cited 2024 Mar 10];287:2288–94. Available from: http://www.jbc.org/article/S0021925820531906/fulltext
97. Coe K, Bostan H, Rolling W, Turner-Hissong S, Macko-Podgórni A, Senalik D, et al. Population genomics identifies genetic signatures of carrot domestication and improvement and uncovers the origin of high-carotenoid orange carrots. Nat Plants [Internet]. 2023 [cited 2024 Mar 13];9:1643–58. Available from: https://www.nature.com/articles/s41477-023-01526-6
98. Maia ACD, Reis LK, Navarro DM do AF, Aristone F, Colombo CA, Carreño-Barrera J, et al. Chemical ecology of Cyclocephala forsteri (Melolonthidae), a threat to macauba oil palm cultivars (Acrocomia aculeata, Arecaceae). J Appl Entomol. 2019;144(1–2):33–40. https://doi.org/10.1111/jen.12699
99. de Araújo VCR, Silva GA, Ramos RS, Júnior PAS, Pereira RR, Motoike SY, et al. Distribution and attack of pineapple mealybug to macauba palm Acrocomia aculeata. Int J Trop Insect Sci. 2021;41(4):2765–73. https://doi.org/10.1007/s42690-021-00456-0
100. Wang L, Lee M, Ye B, Yue GH. Genes, pathways and networks responding to drought stress in oil palm roots. Sci Reports [Internet]. 2020 [cited 2024 Mar 14];10:1–13. Available from: https://www.nature.com/articles/s41598-020-78297-z
101. Suraninpong P, Thongkhao K, Azzeme AM, Suksa-Ard P. Monitoring Drought Tolerance in Oil Palm: Choline Monooxygenase as a Novel Molecular Marker. Plants (Basel). 2023;12(17):3089. https://doi.org/10.3390/plants12173089 PMID: 37687336
102. Golldack D, Lüking I, Yang O. Plant tolerance to drought and salinity: stress regulating transcription factors and their functional significance in the cellular transcriptional network. Plant Cell Rep. 2011;30(8):1383–91. https://doi.org/10.1007/s00299-011-1068-0 PMID: 21476089
103. Gautier M, Gharbi K, Cezard T, Foucaud J, Kerdelhué C, Pudlo P, et al. The effect of RAD allele dropout on the estimation of genetic variation within and between populations. Mol Ecol. 2013;22(11):3165–78. https://doi.org/10.1111/mec.12089 PMID: 23110526
104. Ahrens CW, Rymer PD, Stow A, Bragg J, Dillon S, Umbers KDL, et al. The search for loci under selection: trends, biases and progress. Mol Ecol. 2018;27(6):1342–56. https://doi.org/10.1111/mec.14549 PMID: 29524276
105. Arnold B, Corbett-Detig RB, Hartl D, Bomblies K. RADseq underestimates diversity and introduces genealogical biases due to nonrandom haplotype sampling. Mol Ecol. 2013;22(11):3179–90. https://doi.org/10.1111/mec.12276 PMID: 23551379
106. Aires GCM, de Carvalho Junior RN. Potential of Supercritical Acrocomia aculeata Oil and Its Technology Trends. Appl Sci [Internet]. 2023 [cited 2024 Mar 18];13:8594. Available from: https://www.mdpi.com/2076-3417/13/15/8594/htm
107. Vargas-Carpintero R, Hilger T, Tiede K, Callenius C, Mössinger J, Souza RF, et al. A Collaborative, Systems Approach for the Development of Biomass-Based Value Webs: The Case of the *Acrocomia* Palm. Land [Internet]. 2022 [cited 2024 Mar 14];11:1748. Available from: https://www.mdpi.com/2073-445X/11/10/1748/htm
108. Bertão N. Acelen dá início a projeto para uso da macaúba como fonte de energia. Globo Rural Negócios. 2023.
109. Poland JA, Brown PJ, Sorrells ME, Jannink J-L. Development of high-density genetic maps for barley and wheat using a novel two-enzyme genotyping-by-sequencing approach. PLoS One. 2012;7(2):e32253. https://doi.org/10.1371/journal.pone.0032253 PMID: 22389690
110. Andrews S. FastQC: A Quality Control Tool for High Throughput Sequence Data. 2010.
111. Catchen J, Hohenlohe PA, Bassham S, Amores A, Cresko WA. Stacks: an analysis tool set for population genomics. Mol Ecol. 2013;22(11):3124–40. https://doi.org/10.1111/mec.12354 PMID: 23701397

112. Danecek P, Auton A, Abecasis G, Albers CA, Banks E, DePristo MA, et al. The variant call format and VCFtools. Bioinformatics. 2011;27(15):2156–8. https://doi.org/10.1093/bioinformatics/btr330 PMID: 21653522

113. Manel S, Andrello M, Henry K, Verdelet D, Darracq A, Guerin P-E, et al. Predicting genotype environmental range from genome-environment associations. Mol Ecol. 2018;27(13):2823–33. https://doi.org/10.1111/mec.14723 PMID: 29772088

114. Flanagan SP, Jones AG. Constraints on the FST-Heterozygosity Outlier Approach. J Hered. 2017;108(5):561–73. https://doi.org/10.1093/jhered/esx048 PMID: 28486592

115. R Core Team. R Development Core Team. R A Lang. Environ. Stat. Comput. 2016.

116. Beaumont MA, Nichols RA. Evaluating loci for use in the genetic analysis of population structure. Proc R Soc London Ser B Biol Sci [Internet]. 1996 [cited 2024 Mar 22];263:1619–26. Available from: https://royalsocietypublishing.org/doi/10.1098/rspb.1996.0237

117. Cockerham CC, Weir BS. Estimation of gene flow from F-statistics. Evolution. 1993;47(3):855–63. https://doi.org/10.1111/j.1558-5646.1993.tb01239.x PMID: 28567899

118. Luu K, Bazin E, Blum MGB. pcadapt: an R package to perform genome scans for selection based on principal component analysis. Mol Ecol Resour. 2017;17(1):67–77. https://doi.org/10.1111/1755-0998.12592 PMID: 27601374

119. Frichot E, Schoville SD, Bouchard G, François O. Testing for associations between loci and environmental gradients using latent factor mixed models. Mol Biol Evol. 2013;30(7):1687–99. https://doi.org/10.1093/molbev/mst063 PMID: 23543094

120. Fick SE, Hijmans RJ. WorldClim 2: new 1-km spatial resolution climate surfaces for global land areas. Int J Climatol. 2017;37(12):4302–15. https://doi.org/10.1002/joc.5086

121. Frichot E, Mathieu F, Trouillon T, Bouchard G, François O. Fast and efficient estimation of individual ancestry coefficients. Genetics. 2014;196(4):973–83. https://doi.org/10.1534/genetics.113.160572 PMID: 24496008

122. Frichot E, François O. LEA: An R package for landscape and ecological association studies. Methods Ecol Evol. 2015;6(8):925–9. https://doi.org/10.1111/2041-210x.12382

123. Götz S, García-Gómez JM, Terol J, Williams TD, Nagaraj SH, Nueda MJ, et al. High-throughput functional annotation and data mining with the Blast2GO suite. Nucleic Acids Res. 2008;36(10):3420–35. https://doi.org/10.1093/nar/gkn176 PMID: 18445632
